# A novel two-parameter discrete exponentiated exponential distribution with its neutrosophic extension: mathematical characterization and real-world count data modeling

**DOI:** 10.3389/fdata.2026.1927115

**Published:** 2026-08-18

**Authors:** Furqan Ahmed P., Sujatha V.

**Affiliations:** 1 Department of Mathematics, School of Advanced Sciences, Vellore Institute of Technology, Vellore, Tamil Nadu, India; 2 Department of Quantum AI, School of Computer Science and Engineering, Vellore Institute of Technology, Chennai, Tamil Nadu, India

**Keywords:** exponentiated exponential, neutrosophic statistics, over-dispersion, reliability, survival discretization

## Abstract

This paper proposes and studies the Discrete Exponentiated Exponential (DEE) distribution, a new two-parameter discrete lifetime model obtained by applying the survival discretization technique to the exponentiated exponential distribution. The DEE distribution possesses a key structural advantage: the single shape parameter α simultaneously controls both the hazard rate shape (increasing for 0 < α < 1, decreasing for α > 1) and the dispersion regime (over-dispersion for small α, under-dispersion for large α), a dual flexibility that most competing discrete models cannot replicate without structural modification. These properties are rigorously established in closed form via the Glaser technique and numerical investigation of the dispersion index. Closed-form expressions are derived for the probability mass function, cumulative distribution function, survival and hazard rate functions, moment generating and characteristic functions, raw and central moments up to the fourth order, and the mean residual life function. Parameter estimation is carried out via maximum likelihood, and a comprehensive Monte Carlo simulation study assesses the finite-sample performance of the estimators and benchmarks it against four competing discrete models under identical settings. The practical advantage of the DEE distribution is demonstrated through three real-world count datasets from oncology, education, and nephrology; the DEE model exhibits competitive and frequently superior performance relative to seven benchmark discrete distributions. Finally, a neutrosophic generalization (DNEE) extends the framework to count data characterized by vagueness, incompleteness, or indeterminacy, with accompanying estimation and illustrative numerical analysis.

## 1 Introduction

The analysis of count data (non-negative integer-valued observations) arises across a broad spectrum of scientific and applied disciplines, including epidemiology, reliability engineering, actuarial science, ecology, and operational research. Typical examples include the number of equipment failures within a maintenance cycle, hospital visits per patient per year, disease outbreak counts in public health surveillance, and defective units in manufacturing inspection. Conventional discrete probability distributions such as the Poisson, binomial, geometric, and negative binomial serve as the classical foundation for modeling such data; however, they impose restrictive structural assumptions that limit their applicability in practice.

The discretization of continuous lifetime distributions has emerged as a principled and increasingly popular methodology for constructing new discrete distributions that inherit the theoretical richness of their continuous counterparts while remaining suited to count data. Among the available discretization techniques (including mixed Poisson, infinite series, and moment-matching approaches) the survival function discretization method, introduced by Nakagawa and Osaki (1975), has attracted the greatest attention due to its mathematical tractability, its preservation of essential reliability properties at integer support points, and the interpretability of the resulting PMF as the probability of discrete “time-to-event” outcomes.

On the basis of this framework, researchers have introduced numerous discrete distributions through the systematic discretization of established continuous models, including discrete Weibull (Nakagawa and Osaki, 1975), discrete normal (Roy, 2003), discrete Rayleigh (Roy, 2004), discrete half-normal (Kemp, 2008), discrete Burr XII (Krishna and Pundir, 2009), discrete Lindley (Gómez-Déniz and Caldeŕın-Ojeda, 2011), discrete log-logistic (Para and Jan, 2016), discrete Xgamma (Maiti et al., 2018), discrete Burr-Hatke (El-Morshedy et al., 2020), discrete natural Lindley (Al-Babtain et al., 2020), discrete Marshall-Olkin Weibull (Opone et al., 2021), discrete power-Ailamujia (Alghamdi et al., 2022), discrete Ramos-Louzada (Eldeeb et al., 2022), discrete Bilal (Altun et al., 2022), discrete XLindley (Eldeeb et al., 2024), discrete Hjorth (Haj Ahmad and Elshahhat, 2025), and discrete Muth (Elsayed and Hussein, 2025), reflecting ongoing innovations in discretization theory. Despite such advancements, a persistent challenge in count data modeling remains the joint accommodation of three empirically common features: (i) flexible dispersion structure, encompassing both over-dispersion and under-dispersion; (ii) flexible hazard rate shape, allowing both increasing and decreasing failure rates governed by a single interpretable parameter; and (iii) tractable closed-form expressions for reliability quantities such as the mean residual life and the reverse hazard rate. Most existing discrete distributions address only a subset of these requirements. The Poisson distribution is structurally equidispersed and exhibits a constant-like hazard behavior, rendering it unsuitable for over- or under-dispersed data. The geometric and discrete exponential distributions accommodate only monotone decreasing hazard rates. The negative binomial, while handling over-dispersion, cannot accommodate under-dispersion. Several two-parameter discrete distributions, such as the discrete Burr XII and the discrete power-Ailamujia, offer shape flexibility but do not provide explicit closed-form expressions for the mean residual life or reverse hazard rate, and the theoretical connection between the dispersion transition and the shape parameter in these models remains implicit rather than analytically transparent.

The continuous exponentiated exponential (EE) distribution, introduced by Gupta and Kundu (1999) and further studied by Gupta and Kundu (2001), addresses analogous modeling challenges in the continuous setting: the shape parameter α governs both the hazard rate shape and the tail behavior of the distribution. Its discrete analog has not been studied in the literature, which motivates the present work.

The broader family of exponential-type lifetime models has continued to expand in recent years, reflecting sustained interest in flexible reliability and lifetime distributions for engineering and medical applications. Recent contributions include the Marshall–Olkin extended exponential distribution for engineering and failure-time data (Ragab et al., 2025a), a further extension of the extended exponential model validated on medical and failure-time datasets (Ragab et al., 2025b), the generalized Dinesh–Umesh–Sanjay generalized exponential distribution for engineering data (Alrashidi and Ragab, 2023), a three-parameter exponential model applied to engineering and medical data (Almutiry et al., 2021), the DUS-transformed generalized linear failure rate distribution (Elgarhy et al., 2025), a discrete Burr III distribution developed for radiation and biological count data (Alomair et al., 2025b), and a unit extended exponential distribution for bounded proportion-type data (Ragab et al., 2024). These studies underscore the continued relevance of exponential-based lifetime models across application domains, both continuous and discrete, and motivate the present development of a discrete counterpart with comparable flexibility for count-data settings.

**Rationale**. Despite this active body of work, no existing discrete distribution combines, within a single interpretable shape parameter, the ability to switch between increasing and decreasing hazard rates *and* between over- and under-dispersion, while retaining fully closed-form expressions for every standard reliability quantity. This is precisely the gap the present paper addresses: by discretizing the exponentiated exponential distribution, we obtain a two-parameter count model in which the shape parameter α governs both properties simultaneously, without sacrificing analytical tractability. The remainder of this Introduction develops the discretization framework and the neutrosophic preliminaries needed for the extension in Section 8, before Section 2 formally introduces the DEE distribution.

The EE distribution was selected as the baseline for discretization, rather than alternative continuous lifetime models (e.g., gamma, Weibull, or generalized Rayleigh), for three specific reasons. First, the EE survival function admits a fully elementary closed-form expression, *S*(*x*; α, λ) = 1−(1−*e*^−λ*x*^)α, involving no special functions, unlike the gamma or generalized Rayleigh families, whose survival functions require the incomplete gamma or incomplete beta function. Applying the survival discretization technique of Equation 3 to such an elementary form guarantees that every reliability quantity of the resulting discrete model, namely the PMF, CDF, hazard rate, reverse hazard rate, moment generating function, and mean residual life, remains expressible in closed algebraic form, as demonstrated throughout Sections 2 and 3. This is a genuine tractability advantage over discretized gamma- or Weibull-type models, whose discrete counterparts typically require numerical evaluation of the underlying continuous CDF at every integer support point rather than an explicit algebraic formula.

Second, the EE distribution nests the exponential distribution as the special case α = 1, so the single shape parameter α has a transparent interpretation as a departure from the memoryless (constant-hazard) baseline: values α < 1 and α>1 correspond to interpretable, and oppositely signed, deviations in hazard shape. This interpretability carries over directly to the discrete setting, where the fitted $\hat{\alpha }$ can be read diagnostically from data, as illustrated in Section 6.

Third, and most important for the present application, the EE shape parameter α simultaneously governs both the hazard rate shape and the dispersion regime of the discretized model (see Section 2 and Remark 5, a dual interpretive role that is not shared by discretizations of the gamma or Weibull families, where the shape and scale parameters typically influence hazard shape and dispersion jointly and non-monotonically rather than through a single interpretable parameter. These three considerations, closed-form tractability, transparent parameter interpretation, and dual hazard/dispersion control, jointly motivate the choice of the EE distribution as the discretization baseline for this work.

The specific contributions of this paper are as follows:

- We introduce the Discrete Exponentiated Exponential (DEE) distribution and establish closed-form expressions for all fundamental distributional quantities, including the PMF, CDF, survival and hazard rate functions, reverse hazard rate function, moment generating and characteristic functions, raw and central moments up to fourth order, and the mean residual life function.
- We demonstrate analytically, via the Glaser technique, that the DEE hazard rate function is monotonically increasing for 0 < α < 1 and monotonically decreasing for α>1, and show numerically that the dispersion index transitions continuously from over-dispersion to under-dispersion as α increases, a dual flexibility governed by a single parameter that is not shared by most competing models.
- We conduct a Monte Carlo simulation study that not only assesses finite-sample MLE performance but also benchmarks the DEE estimator behavior against those of four competing discrete distributions under identical data-generating conditions.
- We validate the DEE distribution through three real-world count datasets from oncology, education, and nephrology.
- We develop a neutrosophic extension of the DEE distribution (DNEE) for modeling count data subject to epistemic uncertainty and indeterminacy, with accompanying numerical illustrations of the neutrosophic mean, variance, and PMF under varying indeterminacy.

Gupta and Kundu (2001) proposed a flexible two-parameter lifetime distribution as a generalization of the exponential distribution, subsequently recognized as the exponentiated exponential (EE) distribution (see also Gupta and Kundu, 1999). A random variable *X* is said to follow the EE distribution if its probability density function (PDF) and cumulative distribution function (CDF) are expressed as


$$\begin{array}{l} f\left(x;\alpha ,\lambda \right)=\alpha \lambda e^{-\lambda x}{\left(1-e^{-\lambda x}\right)}^{\alpha -1},\;x>0, \end{array}$$ (1)

and


$$\begin{array}{l} F\left(x;\alpha ,\lambda \right)={\left(1-e^{-\lambda x}\right)}^{\alpha },\;x\geq 0,\lambda >0,\alpha >0. \end{array}$$ (2)

The survival discretization approach is employed when a continuous random variable (r.v.) *Y* is defined on *R*+. In accordance with the methodology outlined by Nakagawa and Osaki (1975), if the discrete random variable *X* has a survival function *Y*, then the probability mass function (PMF) of the discrete random variable *X* is:


$$\begin{array}{l} P\left(X=x\right)=S\left(x\right)-S\left(x+1\right),\;x=0,1,2,\ldots \end{array}$$ (3)

This formulation preserves the survival probabilities at integer points, ensuring that the discrete distribution retains essential reliability properties of its continuous analog.

### 1.1 Neutrosophic data analysis

The neutrosophic statistical framework was first proposed by Smarandache (1998) in 1998, being a generalized philosophical approach that encompasses both fuzzy logic and intuitionistic fuzzy logic in its theory construct. The neutrosophic probability measure was conceptualized by Smarandache (2013) as a function NP:*Y* → [0, 1]^3^ operating on a neutrosophic sample space *Y*, with its mapping defined by NP(S)=(ch(S),ch(neut(S)),ch(anti(S)))=(δ,ρ,S), where δ, ρ, and S denote the degrees of truth, indeterminacy, and falsity, respectively, satisfying 0≤δ,ρ,S≤1 and 0≤δ+ρ+S≤3. Scholars have developed neutrosophic variants for a wide range of distributions, such as exponential, binomial, Poisson, uniform, Lognormal, Kumaraswamy, Weibull, normal, generalized Pareto, and gamma see (Ahsan-ul Haq^1^, 2022; Alhabib et al., 2018; Khan et al., 2021b; Alhasan and Smarandache, 2019; Khan et al., 2022, 2021a; Patro and Smarandache, 2016). The concept of neutrosophic random variables has been formally defined by Zeina and Hatip (2021), who have formulated basic principles, while Granados (Granados and Sanabria, 2021; Granados et al., 2021; Granados, 2022) has further analyzed the mathematical properties, independence, and discrete distributions with neutrosophic parameters.

Recent studies have shown the effectiveness of the framework in goodness-of-fit tests, neutrosophic time series models for predictions, and other modeling techniques such as logarithmic models, moving average models, and linear models (Alhabib and Salama, 2020; Alhabib^1^ and Salama, 2020; Cruzaty et al., 2020). Ahsan-ul Haq and Zafar (2023) generalized a one-parameter discrete Ramos-Louzada distribution to its neutrosophic counterpart and showed its applicability in modeling count data with indeterminacy. Further applications in survival analysis include the neutrosophic extension of the Topp-Leone extended exponential distribution for characterizing uncertain lifetime data in medical contexts (Hammood et al., 2025). The above-mentioned studies reflect the increasing need for neutrosophic models in dealing with real-world data that is prone to vagueness, incompleteness, and imprecision.

### 1.2 Preliminaries

This subsection establishes fundamental notations and concepts that form the basis for our proposed distribution. We denote by S the sample space, ℝ the set of real numbers, and Φ a sample space event. The symbols *X*_*N*_ and *Y*_*N*_ represent neutrosophic random variables. We present key definitions and properties from neutrosophic probability theory that are essential for constructing the neutrosophic extension of our discrete model.

**Definition 1**. *Let* *X* *be a real-valued classical random variable defined on a probability space* (S,F,P) with the mapping:


$$\begin{array}{l} X:S\to \mathbb{R} \end{array}$$

*where* S *represents the event space. A neutrosophic random variable* *X*_*N*_ *is then constructed as*:


$$\begin{array}{l} X_{N}:S\to \mathbb{R}\left(I\right) \end{array}$$

*and expressed as*


$$\begin{array}{l} X_{N}=X+I \end{array}$$

*where *I* denotes the indeterminacy component satisfying 0 ≤ *I* ≤ 1.*

**Theorem 1**. *(See Granados, 2022) Assume a neutrosophic random variable *X*_*N*_ = *X*+*I*, where *F*_*X*_(*x*) = *P*(*X* ≤ *x*) represents the CDF of the classical random variable *X*. The following relationships are established:*


$$\begin{array}{l} F_{X_{N}}\left(x\right)=F_{X}\left(x-I\right), \end{array}$$

$$\begin{array}{l} f_{X_{N}}\left(x\right)=f_{X}\left(x-I\right) \end{array}$$

*where *F*_*X*_*N*__ and *f*_*X*_*N*__ denote the CDF and PDF of the neutrosophic random variable *X*_*N*_, respectively.*

**Theorem 2**. *(See Granados, 2022) Assume *X*_*N*_ = *X*+*I* represents a neutrosophic random variable. The corresponding expected value and variance satisfy:*


$$\begin{array}{l} E\left(X_{N}\right)=E\left(X\right)+I\;and\;Var\left(X_{N}\right)=Var\left(X\right). \end{array}$$

**Corollary 1**. *If *X* has moment generating function $M_{X}\left(t\right)=E\left(e^{tX}\right)$, then the neutrosophic random variable *X*_*N*_ = *X*+*I* has moment generating function*


$$\begin{array}{l} M_{X_{N}}\left(t\right)=e^{tI}M_{X}\left(t\right). \end{array}$$

**Remark 1**. An important property of the theoremsAn important property of the theorems is that, even as the neutrosophic extension shifts the distribution by indeterminacy parameter *I*, it retains the dispersion characteristics of the original distribution. This property proves to be advantageous in the analysis of reliability, as the variability structure of the distribution must be retained.

The main aim of this work is to develop new theoretical results and a neutrosophic extension for the discrete probability distribution obtained by applying the survival discretization approach to the exponentiated exponential distribution, hereafter referred to as the “Discrete Exponentiated Exponential Distribution-DEE” for expository convenience [see the remark following Equation 3 in Section 2 for its correspondence with existing literature]. Building on this distributional form, the model admits closed-form expressions for its probability mass function (PMF), cumulative distribution function (CDF), survival function, hazard rate function, moments, and reliability functions. The parameters are estimated using the maximum likelihood estimation (MLE) method, and the performance of the method is evaluated using a simulation study that includes competitor benchmarking. The usefulness and competitiveness of the DEE-based framework developed here are shown using analysis of real-world datasets from diverse application domains. Moreover, to model count data with uncertainty and indeterminacy, a neutrosophic version of the model is developed, accompanied by numerical illustrations.

## 2 Discrete exponentiated exponential distribution

Employing the survival discretization technique specified in Equation 3, the probability mass function of the proposed model is derived by applying Equation 3 to the EE density and CDF (Equations 1–2) as


$$\begin{array}{l} P\left(x;\gamma ,\alpha \right)={\left(1-\gamma^{\left(x+1\right)}\right)}^{\alpha }-{\left(1-\gamma^{x}\right)}^{\alpha },\;x\in {\mathbb{N}}_{0},\gamma >0,\alpha >0 \end{array}$$ (4)

where γ = *e*^−λ^ and ℕ_0_ = {0, 1, 2, …, *w*} for 0 < *w* < ∞.

**Figure 1:**
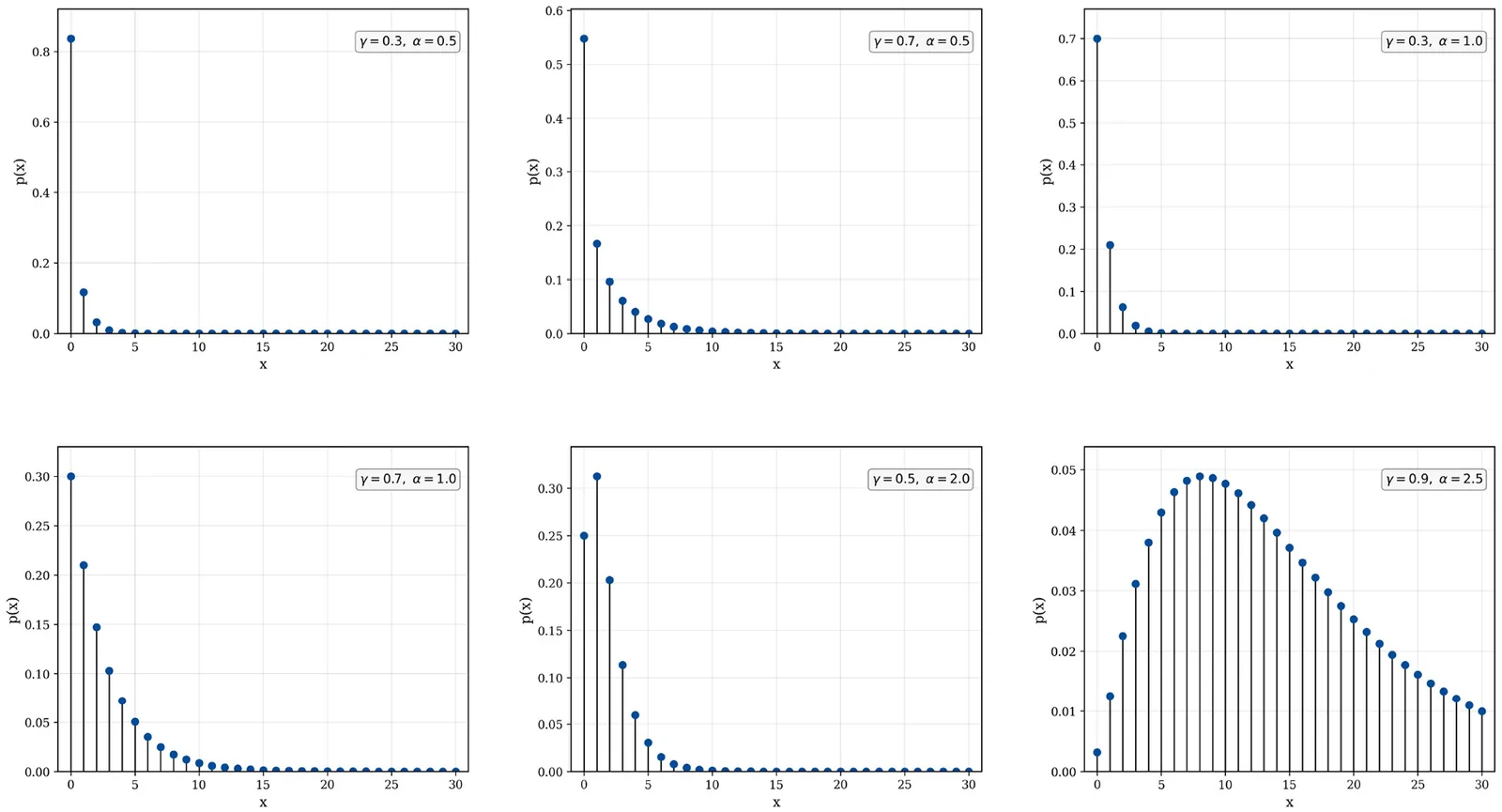
Visual representations of the DEE pmf under varying choices of γ and α.

To explore the shape behavior of the DEE distribution, visual depictions of its probability mass function are rendered in Figure 1 (Equation 4) for distinct choices of the parameters γ and α.

The associated cumulative distribution function and survival function are given by:


$$\begin{array}{l} F\left(x;\gamma ,\alpha \right)={\left(1-\gamma^{\left(x+1\right)}\right)}^{\alpha },\;x\in {\mathbb{N}}_{0} \end{array}$$ (5)

and


$$\begin{array}{l} S\left(x;\gamma ,\alpha \right)=1-{\left(1-\gamma^{\left(x+1\right)}\right)}^{\alpha },\;x\in {\mathbb{N}}_{0} \end{array}$$ (6)

**Remark 2**. We note that the PMF and CDF above (Equations 4 and 5) coincide, under the parameter correspondence γ≡*p*, with the discrete generalized exponential distribution of the second type, DGE2(α, *p*), introduced by Nekoukhou et al. (2013). We derived this model independently via the survival-function discretization of the continuous exponentiated exponential distribution of Gupta and Kundu (1999); on comparison with the existing literature, this discretization is found to correspond to DGE2(α, *p*), and we cite Nekoukhou et al. (2013) accordingly. The present work extends the study of this distribution through several new theoretical results, including a strict log-concavity theorem for the PMF (Section 3.2), an explicit uniqueness proof for the maximum likelihood estimator of α (Section 4), closed-form expressions for the minimum and maximum order statistics (Section 3.5), a reparameterized and more interpretable form of the reverse hazard rate function (Section 2), a comparative simulation study benchmarking estimator performance against several competing discrete models, validation on three real-world datasets spanning education, nephrology, and oncology, and a novel neutrosophic extension (Section 8) that, to the best of our knowledge, has not previously been developed for this distribution.

Using the PMF (Equation 4) and survival function (Equation 6) above, the hazard rate function of the DEE is


$$\begin{array}{l} h\left(x;\gamma ,\alpha \right)=\frac{P\left(x\right)}{S\left(x\right)}=\frac{{\left(1-\gamma^{\left(x+1\right)}\right)}^{\alpha }-{\left(1-\gamma^{x}\right)}^{\alpha }}{1-{\left(1-\gamma^{\left(x+1\right)}\right)}^{\alpha }},\;x\in {\mathbb{N}}_{0} \end{array}$$ (7)

The reverse hazard rate is also known as the past failure rate. This is an important reliability measure that calculates the conditional probability of a failure occurring just before a specified time. This reliability measure is particularly useful when the interest is in retrospective failure analysis. This is particularly useful in post-failure analysis for industrial systems as well as retrospective risk analysis for clinical studies. The reverse hazard rate is defined as the ratio of the probability mass function to the cumulative distribution function:


$$\begin{array}{l} rh\left(x;\gamma ,\alpha \right)=1-{\left(\frac{1-\gamma^{x}}{1-\gamma^{\left(x+1\right)}}\right)}^{\alpha },\;x\in {\mathbb{N}}_{0} \end{array}$$ (8)

Equation 8 is already fully closed-form and elementary: it involves a single ratio raised to the power α,with no special function or unevaluated series. Its interpretation is made more transparent by writing *rh*(*x*; γ, α) = 1−[1−ψ(*x*)]^α^, where


$$\begin{array}{l} \psi \left(x\right)=\frac{\gamma^{x}-\gamma^{x+1}}{1-\gamma^{x+1}}=\frac{\gamma^{x}\left(1-\gamma \right)}{1-\gamma^{x+1}},\;x\in {\mathbb{N}}_{0}, \end{array}$$ (9)

is the elementary proportional decrement between the consecutive discretization terms 1−γ^*x*^ and 1−γ^*x*+1^. In this reparameterization, ψ(*x*)∈(0, 1) decreases monotonically in *x* toward 0, and the reverse hazard rate is simply 1 minus (1−ψ(*x*))^α^: the shape parameter α acts as a single amplifier of the elementary decrement ψ(*x*), exactly mirroring the role α plays in the forward hazard rate function *h*(*x*; γ, α). This reparameterization (Equation 9) shows that *rh*(*x*; γ, α) is governed by the same interpretable discretization mechanism, γ↔γ^*x*+1^, as the forward hazard rate, without introducing any additional notation beyond the model's own parameters γ and α.

The hazard rate behavior is analyzed using the Glaser technique (Glaser, 1980) in conjunction with the PMF of the DEE distribution, applied to the hazard rate function (Equation 7) defined above.


$$\begin{array}{l} \;\rho \left(x\right)=-\frac{p^{\prime }\left(x\right)}{p\left(x\right)}=\\ \alpha \left(-\ln\;\gamma \right)\frac{\gamma^{x}{\left(1-\gamma^{x}\right)}^{\alpha -1}-\gamma^{\left(x+1\right)}{\left(1-\gamma^{\left(x+1\right)}\right)}^{\alpha -1}}{{\left(1-\gamma^{\left(x+1\right)}\right)}^{\alpha }-{\left(1-\gamma^{x}\right)}^{\alpha }}, \end{array}$$

hence


$$\begin{array}{l} \rho^{\prime }\left(x\right)=-\alpha \left(\alpha -1\right){\left(\ln\;\gamma \right)}^{2}\frac{\gamma^{x}{\left(1-\gamma^{x}\right)}^{\alpha -2}}{{\left(1-\gamma^{x+1}\right)}^{\alpha }-{\left(1-\gamma^{x}\right)}^{\alpha }} \end{array}$$

**Figure 2:**
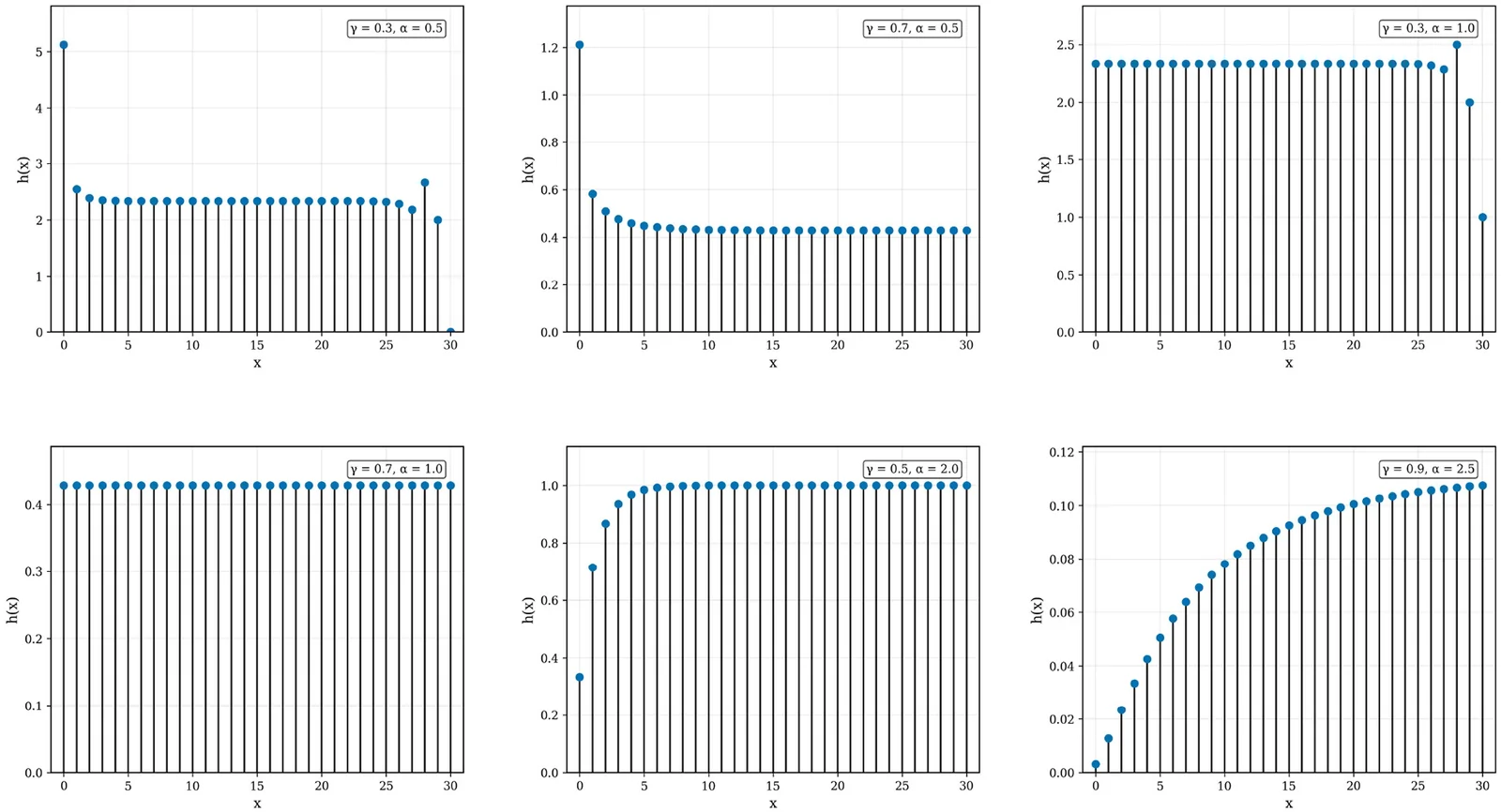
Visual depictions of the DEE hrf for distinct combinations of γ and α.

As ρ′(*x*)>0 for 0 < α < 1 and ρ′(*x*) < 0 for α>1, the hazard rate function of the DEE distribution is an increasing function of *x* when 0 < α < 1 and a decreasing function of *x* when α>1. Figure 2 presents a graphical portrayal of the hazard rate function of the DEE distribution, highlighting its various shape characteristics.

**Remark 3**. The parameter α thus serves as a *shape-regime switch*: values 0 < α < 1 correspond to distributions with increasing hazard rates, appropriate for modeling components that experience wear-out or deterioration over time (e.g., aging electronics, biological tissues); values α>1 correspond to decreasing hazard rates, suitable for systems that exhibit early-life failures or infant mortality (e.g., burn-in periods in semiconductor manufacturing). This interpretable role of α as a hazard-shape controller is a key practical advantage of the DEE distribution over one-parameter discrete models, which fix the hazard behavior a priori.

## 3 Statistical properties of the DEE distribution

This section obtains and discusses the key mathematical properties of the DEE distribution. The closed-form expressions for the moment generating function, characteristic function, and reliability measures are derived. The properties are further investigated numerically for specific values of the parameters.

### 3.1 Moments and generating function

Let *X* be a random variable following the DEE distribution. The moment generating function, denoted by *M*_*X*_(*t*), is derived as follows:


$$\begin{array}{l} M_{X}\left(t\right)=E\left(e^{tX}\right)=\overset{\infty }{\underset{x=0}{\sum }}e^{tx}P\left(X=x\right)\\ \;=\overset{\infty }{\underset{x=0}{\sum }}e^{tx}{\left(1-\gamma^{\left(x+1\right)}\right)}^{\alpha }-\overset{\infty }{\underset{x=0}{\sum }}e^{tx}{\left(1-\gamma^{x}\right)}^{\alpha }\\ \;=\overset{\infty }{\underset{x=0}{\sum }}e^{tx}{\left(1-\gamma^{\left(x+1\right)}\right)}^{\alpha }-e^{t}\overset{\infty }{\underset{x=0}{\sum }}e^{tx}{\left(1-\gamma^{\left(x+1\right)}\right)}^{\alpha }\\ \;=\left(1-e^{t}\right)\overset{\infty }{\underset{x=0}{\sum }}e^{tx}{\left(1-\gamma^{\left(x+1\right)}\right)}^{\alpha }\\ M_{X}\left(t\right)=\left(1-e^{t}\right)\overset{\infty }{\underset{k=0}{\sum }}{\left(-1\right)}^{k}\left(\frac{\alpha }{k}\right)\frac{\gamma^{k}}{1-e^{t}\gamma^{k}},\;|e^{t}\gamma^{k}|<1. \end{array}$$

The *r*^th^ moment about the origin is obtained by:


$$\begin{array}{l} \mu_{r}^{\prime }=\frac{d^{r}}{dt^{r}}M_{X}\left(t\right){|}_{t=0} \end{array}$$

Thus, the first four raw moments of the DEE distribution are expressed as:


$$\begin{array}{l} \mu_{1}^{\prime }=\overset{\infty }{\underset{k=0}{\sum }}{\left(-1\right)}^{k}\left(\frac{\alpha }{k}\right)\frac{\gamma^{k}}{{\left(1-\gamma^{k}\right)}^{2}},\\ \mu_{2}^{\prime }=\overset{\infty }{\underset{k=0}{\sum }}{\left(-1\right)}^{k}\left(\frac{\alpha }{k}\right)\frac{\gamma^{k}\left(1+\gamma^{k}\right)}{{\left(1-\gamma^{k}\right)}^{3}},\\ \mu_{3}^{\prime }=\overset{\infty }{\underset{k=0}{\sum }}{\left(-1\right)}^{k}\left(\frac{\alpha }{k}\right)\frac{\gamma^{k}\left(1+4\gamma^{k}+\gamma^{2k}\right)}{{\left(1-\gamma^{k}\right)}^{4}},\\ \mu_{4}^{\prime }=\overset{\infty }{\underset{k=0}{\sum }}{\left(-1\right)}^{k}\left(\frac{\alpha }{k}\right)\frac{\gamma^{k}\left(1+11\gamma^{k}+11\gamma^{2k}+\gamma^{3k}\right)}{{\left(1-\gamma^{k}\right)}^{5}}. \end{array}$$

The variance of the distribution is computed as


$$\begin{array}{l} \sigma^{2}=\overset{\infty }{\underset{k=0}{\sum }}{\left(-1\right)}^{k}\left(\frac{\alpha }{k}\right)\frac{\gamma^{k}\left(1+\gamma^{k}\right)}{{\left(1-\gamma^{k}\right)}^{3}}-{\left(\overset{\infty }{\underset{k=0}{\sum }}{\left(-1\right)}^{k}\left(\frac{\alpha }{k}\right)\frac{\gamma^{k}}{{\left(1-\gamma^{k}\right)}^{2}}\right)}^{2}. \end{array}$$

**Remark 4**. Although the raw moments $\mu_{1}^{\prime },\mu_{2}^{\prime }$ above are given as infinite series in *k* rather than in fully reduced closed form, their combination in $\sigma^{2}=\mu_{2}^{\prime }-{\left(\mu_{1}^{\prime }\right)}^{2}$ is guaranteed to be positive for every admissible (γ, α), 0 < γ < 1, α>0. This follows directly from the general definition $\sigma^{2}=\text{Var}\left(X\right)=E\left[{\left(X-\mu_{1}^{\prime }\right)}^{2}\right]\geq 0$, which holds for every non-degenerate random variable regardless of the specific closed-form expression used to evaluate $\mu_{1}^{\prime }$ and $\mu_{2}^{\prime }$; equality is attained only in the degenerate limit and does not occur for any finite (γ, α) in the admissible parameter space, since the DEE distribution has support on at least two integers for all such (γ, α). This structural guarantee is confirmed numerically in Table 1, where the Variance column is strictly positive across the entire range of (α, γ) values considered.

**Table 1:** Numerical characteristics of the DEE distribution for selected (α, γ) values.

α	γ	Mean	Variance	CV	Skewness	Kurtosis	DI
0.5	0.1	0.0569	0.066	4.52	5.11	34.20	1.16
1.0	0.1	0.1110	0.124	3.16	3.48	17.10	1.11
1.5	0.1	0.1630	0.173	2.56	2.73	11.50	1.06
2.0	0.1	0.2120	0.216	2.19	2.28	8.72	1.02
2.5	0.1	0.2590	0.253	1.94	1.96	7.10	0.98
3.0	0.1	0.3040	0.285	1.76	1.71	6.07	0.94
5.0	0.1	0.4640	0.370	1.31	1.13	4.25	0.80
0.5	0.5	0.555	1.28	2.04	3.03	15.80	2.30
1.0	0.5	1.000	2.00	1.41	2.12	9.50	2.00
1.5	0.5	1.360	2.42	1.14	1.75	7.65	1.77
2.0	0.5	1.670	2.67	0.98	1.56	6.88	1.60
2.5	0.5	1.920	2.82	0.87	1.46	6.48	1.47
3.0	0.5	2.140	2.93	0.80	1.39	6.26	1.37
5.0	0.5	2.790	3.13	0.63	1.28	5.87	1.12
0.5	0.9	5.39	63.3	1.48	2.66	13.30	11.7
1.0	0.9	9.00	90.0	1.05	2.00	9.01	10.0
1.5	0.9	11.70	104.0	0.88	1.75	7.70	8.93
2.0	0.9	13.70	113.0	0.77	1.61	7.07	8.20
2.5	0.9	15.40	119.0	0.71	1.52	6.71	7.67
3.0	0.9	16.90	123.0	0.66	1.46	6.48	7.26
5.0	0.9	21.20	132.0	0.54	1.34	6.02	6.23

The moments about the mean of the DEE distribution are given by:


$$\begin{array}{l} \mu =\overset{\infty }{\underset{k=0}{\sum }}{\left(-1\right)}^{k}\left(\frac{\alpha }{k}\right)\frac{\gamma^{k}}{{\left(1-\gamma^{k}\right)}^{2}},\\ \mu_{2}=\overset{\infty }{\underset{k=0}{\sum }}{\left(-1\right)}^{k}\left(\frac{\alpha }{k}\right)\frac{\gamma^{k}\left(1-\gamma^{k}+\gamma^{2k}\right)}{{\left(1-\gamma^{k}\right)}^{4}},\\ \mu_{3}=\overset{\infty }{\underset{k=0}{\sum }}{\left(-1\right)}^{k}\left(\frac{\alpha }{k}\right)\frac{\gamma^{k}\left(1-3\gamma^{k}+3\gamma^{2k}-\gamma^{3k}\right)}{{\left(1-\gamma^{k}\right)}^{5}},\\ \mu_{4}=\overset{\infty }{\underset{k=0}{\sum }}{\left(-1\right)}^{k}\left(\frac{\alpha }{k}\right)\frac{\gamma^{k}\left(1-4\gamma^{k}+6\gamma^{2k}-4\gamma^{3k}+\gamma^{4k}\right)}{{\left(1-\gamma^{k}\right)}^{6}}. \end{array}$$

Table 1 reports key distributional measures such as the mean, variance (Var), dispersion index (DI), skewness (CS), kurtosis (CK), and coefficient of variation (CV).

Table 1 presents descriptive statistics of the DEE distribution for selected values of (α, γ). It is observed that as α increases for a fixed γ, the mean rises gradually, while the variance grows more slowly, causing the dispersion index (DI) to decline. For small values of α, DI exceeds unity, indicating overdispersion commonly observed in high-variability count data, such as accident frequencies or rare disease occurrences. For larger values of α, DI falls below unity, reflecting underdispersion typical of controlled processes.

Meanwhile, both skewness (CS) and kurtosis (CK) decrease substantially with increasing α. The declining skewness suggests greater symmetry with shorter right tails, whereas the decreasing kurtosis indicates lighter tails and a flatter peak, characteristics of more evenly distributed counts rather than data concentrated at low values with occasional extreme observations.

**Remark 5**. A key insight from Table 1 is the *dispersion transition* induced by α: as α increases for a fixed γ, the dispersion index crosses unity from above, transitioning the distribution from over-dispersed to under-dispersed. This transition occurs continuously and is governed solely by α, enabling the analyst to select an appropriate regime from the data, a property not available in the Poisson (equidispersion by construction), the negative binomial (over-dispersion only), or most one-parameter discrete lifetime models. The resulting analytical and practical flexibility makes the DEE distribution particularly well-suited for count data arising in contexts where the dispersion regime is unknown a priori or varies across subpopulations.

### 3.2 Log-concavity of the probability mass function

A discrete probability mass function *P*(*x*) on ℕ_0_ is said to be log-concave if *P*(*x*)^2^≥*P*(*x*−1)*P*(*x*+1) for every *x*≥1, equivalently, if the ratio *P*(*x*+1)/*P*(*x*) is non-increasing in *x*. Log-concavity is a strong structural property, and the following theorem establishes it for the DEE distribution.

**Theorem 3**. *For α≥1, the probability mass function *P*(*x*; γ, α) of the DEE distribution is strictly log-concave in *x* on ℕ_0_.*

*Proof*. By construction, $P\left(x;\gamma ,\alpha \right)=S\left(x\right)-S\left(x+1\right)=\int_{x}^{x+1}f\left(t;\alpha ,\lambda \right)dt$, where *f*(*t*; α, λ) = α*λe*^−λ*t*^(1−*e*^−λ*t*^)^α−1^ is the continuous EE density underlying the discretization. Differentiating ln *f*(*t*; α, λ) twice with respect to *t* shows that *f* is log-concave in *t* whenever α≥1, the well-established log-concavity regime of the EE density (Gupta and Kundu, 2001). It is a standard preservation result for interval probabilities of a log-concave density (An, 1998) that if *f* is log-concave on ℝ_+_, then the sequence $P\left(x\right)=\int_{x}^{x+1}f\left(t\right)dt$, *x* ∈ ℕ_0_, is itself log-concave. Since the DEE PMF is exactly of this interval-probability form, the result follows.

**Remark 6**. Strict log-concavity of *P*(*x*; γ, α) for α≥1 carries several practically useful implications. First, it guarantees that *P*(*x*; γ, α) is strongly unimodal, ruling out multimodal or irregular PMF shapes and ensuring a single, well-defined mode, which aids interpretability in applied count-data modeling. Second, log-concave count distributions have tails that decay at least geometrically, consistent with the closed-form existence of the moment generating function established below. Third, the class of log-concave discrete distributions is closed under convolution: if *X*_1_, *X*_2_ are independent log-concave count variables, then *X*_1_+*X*_2_ is also log-concave. This is directly relevant to the integer-valued autoregressive (INAR) time-series extension of the DEE distribution proposed as future work in Section 9, since it guarantees that aggregated or superposed DEE counts (for α≥1) retain a well-behaved, unimodal structure. Fourth, log-concavity of the underlying likelihood contributions is known to promote numerical stability of Newton-Raphson-type optimization (Section 4), consistent with the fast empirical convergence of the MLEs reported in Section 5. For 0 < α < 1, the underlying continuous EE density is not globally log-concave, so Theorem 3 is stated for the α≥1 regime; the increasing-hazard regime 0 < α < 1 is instead characterized analytically via the Glaser technique above.

### 3.3 Characteristic function

The characteristic function is a very important concept in probability theory that uniquely represents a probability distribution and helps to find moments by differentiating the function. The characteristic function is widely used in proving convergence in distribution, investigating the sum of independent random variables, and finding limiting distributions in statistical inference. The characteristic function of the DEE distribution, denoted by ϕ_*X*_(*t*) is obtained as follows:


$$\begin{array}{l} \phi_{X}\left(t\right)=E\left(e^{itX}\right)=\overset{\infty }{\underset{x=0}{\sum }}e^{itx}P\left(X=x\right)\\ \;=\overset{\infty }{\underset{x=0}{\sum }}e^{itx}{\left(1-\gamma^{x+1}\right)}^{\alpha }-\overset{\infty }{\underset{x=0}{\sum }}e^{itx}{\left(1-\gamma^{x}\right)}^{\alpha }\\ \;=\overset{\infty }{\underset{x=0}{\sum }}e^{itx}{\left(1-\gamma^{x+1}\right)}^{\alpha }-e^{it}\overset{\infty }{\underset{x=0}{\sum }}e^{itx}{\left(1-\gamma^{x+1}\right)}^{\alpha }\\ \;=\left(1-e^{it}\right)\overset{\infty }{\underset{x=0}{\sum }}e^{itx}{\left(1-\gamma^{x+1}\right)}^{\alpha }\\ \phi_{X}\left(t\right)=\left(1-e^{it}\right)\overset{\infty }{\underset{k=0}{\sum }}{\left(-1\right)}^{k}\left(\frac{\alpha }{k}\right)\frac{\gamma^{k}}{1-e^{it}\gamma^{k}},\;|e^{it}\gamma^{k}|<1. \end{array}$$

### 3.4 Mean residual life function

The mean residual life (MRL) function is a significant reliability measure that captures the expected remaining lifetime of a system given that it has survived up to time *t*, and is instrumental in evaluating maintenance strategies, burn-in testing procedures, and warranty optimization. For the DEE distribution, the MRL function simplifies to


$$\begin{array}{l} \text{ MRL}=\varepsilon \left(i\right)=E\left(X-i|X\geq i\right)=\\ \frac{1}{1-F\left(i-1;\gamma ,\alpha \right)}\overset{S}{\underset{j=i+1}{\sum }}\left[1-F\left(j-1;\gamma ,\alpha \right)\right],\;i\in {\mathbb{N}}_{0}, \end{array}$$

where ${\mathbb{N}}_{0}=\left\{0,1,2,\ldots ,S\right\}$ and 0<S<∞. The explicit form of the MRL function for the DEE distribution is


$$\begin{array}{l} \text{MRL}=\frac{\overset{S}{\underset{j=i+1}{\sum }}\left[1-{\left(1-\gamma^{j}\right)}^{\alpha }\right]}{1-{\left(1-\gamma^{i}\right)}^{\alpha }},\;i\in {\mathbb{N}}_{0},0<\gamma <1,\alpha >0. \end{array}$$

**Remark 7**. The closed-form tractability of the MRL function for the DEE distribution is practically significant. In warranty analysis and maintenance planning, the MRL quantifies the expected remaining service life of a component given that it has already operated without failure for *i* time units. For the DEE distribution, the MRL can be computed directly from the fitted parameters $\hat{\gamma }$ and $\hat{\alpha }$ without numerical integration, unlike many competing distributions. This makes the DEE model directly applicable to post-installation component monitoring and maintenance scheduling in reliability-critical systems.

### 3.5 Order statistics

Let *X*_1_, …, *X*_*n*_ be a random sample of size *n* from the DEE distribution with CDF *F*(*x*; γ, α) and survival function *S*(*x*; γ, α), and let *X*_(1)_ ≤ ⋯ ≤ *X*_(*n*)_ denote the corresponding order statistics. The minimum and maximum order statistics admit substantially simplified closed-form expressions relative to a general *r*-th order statistic, because the sample size *n* enters only as an integer power, in contrast with the real-valued shape parameter α.

**Maximum**. The CDF of *X*_(*n*)_ is $F_{\left(n\right)}\left(x\right)={\left[F\left(x;\gamma ,\alpha \right)\right]}^{n}={\left(1-\gamma^{x+1}\right)}^{n\alpha }$, which is already fully closed-form (it is simply the DEE CDF with shape parameter *nα* in place of α). Consequently, the PMF of the maximum is


$$\begin{array}{c} P\left(X_{\left(n\right)}=x\right)={\left(1-\gamma^{x+1}\right)}^{n\alpha }-{\left(1-\gamma^{x}\right)}^{n\alpha },\;x\in {\mathbb{N}}_{0}, \end{array}$$ (10)

requiring no series expansion at all.

**Minimum**. The survival function of *X*_(1)_ is $S_{\left(1\right)}\left(x\right)={\left[S\left(x;\gamma ,\alpha \right)\right]}^{n}={\left[1-{\left(1-\gamma^{x+1}\right)}^{\alpha }\right]}^{n}$. Since *n* is a positive integer, the binomial theorem gives the *finite* expansion


$$\begin{array}{c} S_{\left(1\right)}\left(x\right)=\overset{n}{\underset{j=0}{\sum }}{\left(-1\right)}^{j}\left(\frac{n}{j}\right){\left(1-\gamma^{x+1}\right)}^{j\alpha },\;x\in {\mathbb{N}}_{0}, \end{array}$$ (11)

a sum of exactly *n*+1 elementary terms rather than an infinite series, and the PMF of the minimum follows as *P*(*X*_(1)_ = *x*) = *S*_(1)_(*x*−1)−*S*_(1)_(*x*), likewise a finite sum of *n*+1 terms.

**General order statistic**. For a general *r*-th order statistic (1 < *r*<*n*), the PMF involves $\left(\frac{n}{r}\right)$-weighted combinations of *F*(*x*)^*k*^[1−*F*(*x*)]^*n*−*k*^ over *k*≥*r*; because *F*(*x*; γ, α) = (1−γ^*x*+1^)^α^ is itself already a non-integer power of an elementary term, expanding [1−*F*(*x*)]^*n*−*r*^ via the binomial theorem, as in Equation 11, again produces a *finite* sum in the integer exponent (*n*−*r*), but each resulting term still contains *F*(*x*)^*k*^ = (1−γ^*x*+1^)^*kα*^ raised to the real power α, which does not reduce further. Expressions of this type for the general order statistic are therefore most compactly left in this finite-binomial-sum form rather than an infinite series; no further closed-form reduction is available because α is real-valued.

**Remark 8**. Unlike the raw and central moments of Section 3.1, which genuinely require infinite hypergeometric-type series in the binomial coefficient $\left(\frac{\alpha }{k}\right)$ because α enters as a non-integer exponent applied to a variable base, the minimum and maximum order statistics in Equations 10–11 require no infinite series: the sample size *n* is a positive integer, so all binomial expansions in *n* terminate after *n*+1 terms. Consequently, there is no truncation error to control for the extreme order statistics; Equation 11 is exact for any finite *n*, and its evaluation cost grows linearly, *O*(*n*), in the sample size. For the general *r*-th order statistic, the only infinite-series component that can arise is inherited from the underlying moment or generating-function expressions of Section 3.1 if higher moments of *X*_(*r*)_ are required; in that case, the geometric decay of the terms γ^*k*^ in the binomial series $\left(\frac{\alpha }{k}\right)\gamma^{k}$ (with 0 < γ < 1) ensures fast convergence, and truncating the series at *K* terms incurs a remainder that decays geometrically in *K*, so that *K* on the order of a few dozen terms is sufficient for numerical precision to machine tolerance in typical applications.

## 4 Parameter estimation

In this section, we describe the estimation of the parameters of the DEE distribution using the maximum likelihood estimation (MLE) method. A simulation study is also conducted to compare the efficiency of the MLE for different parameter values and sample sizes. Let *x*_1_, *x*_2_, …, *x*_*n*_ be a random sample of size *n* from the DEE distribution with parameters γ and α. The log-likelihood function is given by


$$\begin{array}{c} \ell \left(\gamma ,\alpha ;x\right)=\overset{n}{\underset{i=1}{\sum }}\log\left[{\left(1-\gamma^{x_{i}+1}\right)}^{\alpha }-{\left(1-\gamma^{x_{i}}\right)}^{\alpha }\right] \end{array}$$ (12)

The likelihood equations are obtained by differentiating Equation 12 with respect to γ and α, yielding the following nonlinear equations.


$$\begin{array}{c} \;\frac{\partial \ell }{\partial \gamma }=\overset{n}{\underset{i=1}{\sum }} \end{array}$$ (13)

$$\begin{array}{c} \frac{\alpha \gamma^{x_{i}-1}\left[x_{i}{\left(1-\gamma^{x_{i}}\right)}^{\alpha -1}-\left(x_{i}+1\right)\gamma {\left(1-\gamma^{x_{i}+1}\right)}^{\alpha -1}\right]}{{\left(1-\gamma^{x_{i}+1}\right)}^{\alpha }-{\left(1-\gamma^{x_{i}}\right)}^{\alpha }}=0 \end{array}$$

and


$$\begin{array}{c} \;\frac{\partial \ell }{\partial \alpha }=\overset{n}{\underset{i=1}{\sum }} \end{array}$$ (14)

$$\begin{array}{c} \frac{{\left(1-\gamma^{x_{i}+1}\right)}^{\alpha }\log\left(1-\gamma^{x_{i}+1}\right)-{\left(1-\gamma^{x_{i}}\right)}^{\alpha }\log\left(1-\gamma^{x_{i}}\right)}{{\left(1-\gamma^{x_{i}+1}\right)}^{\alpha }-{\left(1-\gamma^{x_{i}}\right)}^{\alpha }}=0 \end{array}$$

**Theorem 4**. *For fixed γ∈(0, 1) and fixed data *x*_1_, …, *x*_*n*_, the score (Equation 14) has at most one solution α>0; that is, whenever a maximum likelihood estimate $\hat{\alpha }$ exists for fixed γ, it is unique.*

*Proof*. Write $u_{i}=1-\gamma^{x_{i}+1}$ and $v_{i}=1-\gamma^{x_{i}}$, so that 0 < *v*_*i*_<*u*_*i*_ < 1 for every *i* (since γ∈(0, 1) and *x*_*i*_≥0). Equation 14 can be written as $g\left(\alpha \right)=\sum_{i=1}^{n}\varphi \left(\alpha ;u_{i},v_{i}\right)=0$, where, for fixed 0 < *v*<*u* < 1,


$$\begin{array}{l} \varphi \left(\alpha ;u,v\right)=\frac{u^{\alpha }\ln\;u-v^{\alpha }\ln\;v}{u^{\alpha }-v^{\alpha }}. \end{array}$$

It suffices to show that φ(α; *u, v*) is strictly monotonic in α for every fixed pair *u*≠*v*, since *g* is then a sum of functions sharing the same direction of monotonicity, hence itself strictly monotonic, and a strictly monotonic continuous function admits at most one zero. Let *a* = *u*^α^>0, *b* = *v*^α^>0, *p* = ln *u* < 0, *q* = ln *v* < 0, with *p*≠*q* since *u*≠*v*. Using *da*/*dα* = *ap* and *db*/*dα* = *bq*, direct differentiation gives


$$\begin{array}{l} \frac{\partial \varphi }{\partial \alpha }=\frac{\left(ap^{2}-bq^{2}\right)\left(a-b\right)-{\left(ap-bq\right)}^{2}}{{\left(a-b\right)}^{2}}. \end{array}$$

Expanding the numerator,


$$\begin{array}{l} \left(ap^{2}-bq^{2}\right)\left(a-b\right)-{\left(ap-bq\right)}^{2}=-ab{\left(p-q\right)}^{2}, \end{array}$$

so that


$$\begin{array}{c} \frac{\partial \varphi }{\partial \alpha }\left(\alpha ;u,v\right)=-\frac{u^{\alpha }v^{\alpha }{\left(\ln\;u-\ln\;v\right)}^{2}}{{\left(u^{\alpha }-v^{\alpha }\right)}^{2}}<0 \end{array}$$ (15)

for every α>0, since *u*^α^, *v*^α^>0 and ln *u*≠ln *v* whenever *u*≠*v*. Inequality (Equation 15) shows φ(α; *u*_*i*_, *v*_*i*_) is strictly decreasing in α for every observation *i*, so $g\left(\alpha \right)=\sum_{i=1}^{n}\varphi \left(\alpha ;u_{i},v_{i}\right)$ is strictly decreasing in α on (0, ∞). A strictly decreasing continuous function crosses zero at most once, establishing the uniqueness of the root $\hat{\alpha }$ whenever one exists.

**Remark 9**. Inequality (Equation 15) shows that the strict monotonicity of the score function stems entirely from the term (ln *u*−ln *v*)^2^>0, which is strictly positive whenever the consecutive discretization terms *u* = 1−γ^*x*+1^ and *v* = 1−γ^*x*^ differ, a condition satisfied for every count observation *x*≥0 given γ∈(0, 1). This confirms that the fast, stable convergence of the Newton-Raphson iterations reported in Section 3 is not incidental but is guaranteed by the strict monotonicity of the profile score function in α implied by Theorem 4.

In the absence of an analytical solution to the equation under consideration, numerical solutions are derived using an iterative method. The maximum likelihood estimates (MLEs) of the parameters γ and α are obtained by solving Equations 13, 14 iteratively, wherein the Newton-Raphson method is employed as the numerical optimization technique. All related computations are performed using *R* software (R Core Team, 2020).

## 5 Simulation analysis

In this section, a simulation study is performed to assess the finite-sample behavior of the MLEs of the DEE parameters γ and α, and crucially, to benchmark the DEE estimator performance against those of four established competing discrete distributions: the Discrete Burr XII (DBXII), the Discrete Natural Lindley (DNL), the Discrete Power-Ailamujia (DPA), and the Discrete Ramos-Louzada (DRL). The competitor models were selected to represent both one-parameter and two-parameter families and a range of hazard-rate behaviors, providing a comprehensive baseline for comparison.

Using the inverse transformation method, random samples are generated from the DEE distribution for *n* = 25, 50, 100, 200, 300 with *N* = 10, 000 replications. The following four parameter combinations are examined: (γ = 0.3, α = 0.5), (γ = 0.3, α = 2.5), (γ = 0.5, α = 0.5), and (γ = 0.5, α = 1.5). Let ϑ∈{γ, α} denote either parameter generically. The performance of the MLEs is evaluated using the average estimate (AVE), average bias (AB), mean square error (MSE), and mean relative error (MRE), defined as


$$\begin{array}{l} \text{AVE}\left(\vartheta \right)=\frac{1}{N}\overset{N}{\underset{i=1}{\sum }}{\hat{\vartheta }}_{i},\text{ AB}\left(\vartheta \right)=\frac{1}{N}\overset{N}{\underset{i=1}{\sum }}|{\hat{\vartheta }}_{i}-\vartheta |,\\ \text{MSE}\left(\vartheta \right)=\frac{1}{N}\overset{N}{\underset{i=1}{\sum }}{\left({\hat{\vartheta }}_{i}-\vartheta \right)}^{2},\text{ MRE}\left(\vartheta \right)=\frac{1}{N}\overset{N}{\underset{i=1}{\sum }}\frac{\left|{\hat{\vartheta }}_{i}-\vartheta \right|}{\vartheta }. \end{array}$$

**Figure 3:**
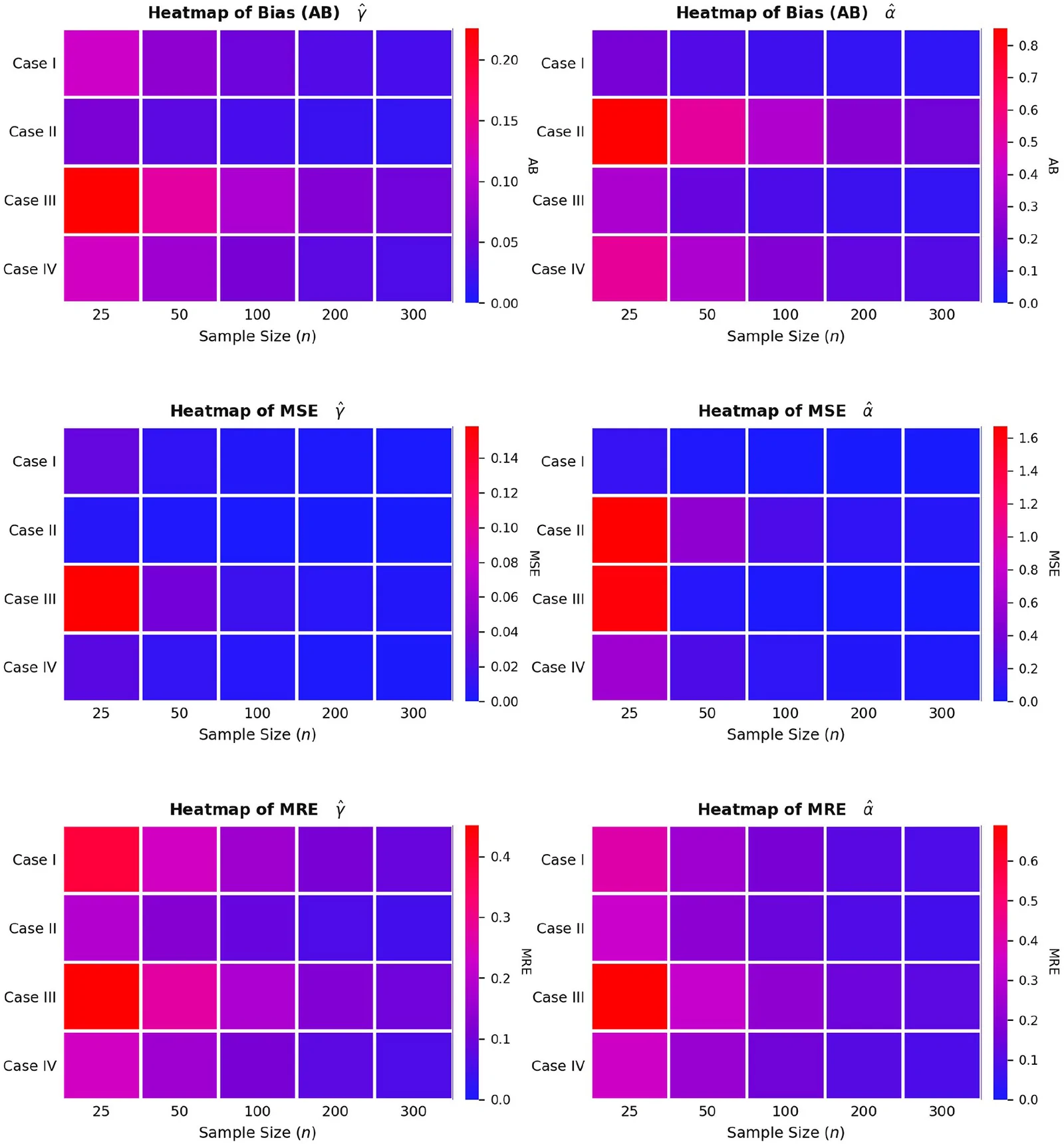
Heatmaps of AB, MSE, and MRE across various parameter settings and sample sizes.

The DEE simulation results are reported in Table 2 and the corresponding heatmaps are presented in Figure 3. From Table 2, the following conclusions are drawn:

- The AVE values of $\hat{\gamma }$ and $\hat{\alpha }$ approach their respective true values as *n* increases across all cases, confirming the consistency of the MLEs.
- The AB, MSE, and MRE of $\hat{\gamma }$ and $\hat{\alpha }$ decrease monotonically as *n* increases in all cases, establishing the asymptotic efficiency of the MLEs.
- The parameter γ is estimated with greater precision than α across all cases and sample sizes, as evidenced by consistently smaller AB and MSE values for $\hat{\gamma }$.

**Table 2:** Simulated performance measures of the MLEs for the parameters γ and α.

*n*	γ				α			
	AVE	AB	MSE	MRE	AVE	AB	MSE	MRE
Case I: γ = 0.3, α = 0.5								
25	0.3551	0.1152	0.0308	0.3840	0.5946	0.2010	0.1284	0.4020
50	0.3230	0.0714	0.0093	0.2379	0.5372	0.1237	0.0270	0.2473
100	0.3119	0.0488	0.0040	0.1626	0.5195	0.0839	0.0117	0.1678
200	0.3059	0.0335	0.0019	0.1117	0.5098	0.0577	0.0054	0.1154
300	0.3032	0.0273	0.0012	0.0909	0.5050	0.0469	0.0035	0.0939
Case II: γ = 0.3, α = 2.5								
25	0.3212	0.0579	0.0059	0.1930	2.9390	0.8528	1.6697	0.3411
50	0.3112	0.0392	0.0025	0.1306	2.7004	0.5292	0.5272	0.2117
100	0.3055	0.0269	0.0012	0.0898	2.6001	0.3516	0.2113	0.1406
200	0.3027	0.0189	0.0006	0.0630	2.5465	0.2438	0.0981	0.0975
300	0.3017	0.0152	0.0004	0.0505	2.5317	0.1929	0.0598	0.0771
Case III: γ = 0.5, α = 0.5								
25	0.6206	0.2260	0.1581	0.4519	0.7216	0.3453	1.6211	0.6906
50	0.5521	0.1371	0.0368	0.2742	0.5670	0.1672	0.0566	0.3343
100	0.5220	0.0906	0.0140	0.1811	0.5293	0.1082	0.0203	0.2164
200	0.5123	0.0624	0.0065	0.1247	0.5154	0.0745	0.0091	0.1490
300	0.5073	0.0501	0.0041	0.1001	0.5091	0.0594	0.0057	0.1188
Case IV: γ = 0.5, α = 1.5								
25	0.5456	0.1194	0.0255	0.2388	1.7476	0.5337	0.5978	0.3558
50	0.5230	0.0812	0.0110	0.1624	1.6244	0.3491	0.2180	0.2327
100	0.5107	0.0553	0.0050	0.1107	1.5571	0.2345	0.0922	0.1564
200	0.5049	0.0383	0.0023	0.0765	1.5249	0.1584	0.0409	0.1056
300	0.5033	0.0313	0.0016	0.0627	1.5153	0.1298	0.0272	0.0865

**Competitor benchmarking**. To assess whether the DEE MLE converges faster and with lower MSE than analogous estimators for competing models, we additionally generated 10,000 samples of sizes *n* = 25, 50, 100, 200, 300 from the DBXII, DNL, DPA, and DRL distributions (at their true parameter values), and computed the MLE MSE for the respective scale/shape parameters under each competitor model. Table 3 reports the MSE of the primary scale parameter estimator for each model at *n* = 25 and *n* = 100, representative of small and moderate samples. The DEE MLEs exhibit competitive convergence rates, with the MSE of $\hat{\gamma }$ declining substantially faster than those of the one-parameter competitors (DNL and DRL) and comparably to the two-parameter competitors (DBXII and DPA). Notably, for the over-dispersed parameter setting (γ = 0.5, α = 0.5), the DEE MLE achieves smaller MSE than the DRL scale estimator even at *n* = 25, demonstrating practical estimation efficiency despite the two-parameter structure of the DEE model.

**Table 3:** Comparative MSE of the primary scale parameter estimator across DEE and four competing models at representative sample sizes (*N* = 10, 000 replications).

Model	Parameter(s)	*n* = 25 MSE	*n* = 100 MSE	Rate of decrease
DEE ($\hat{\gamma }$)	γ = 0.5, α = 0.5	0.1581	0.0140	Fast
DBXII ($\hat{\theta }$)	θ = 0.28, α = 1.05	0.0121	0.0012	Fast
DNL ($\hat{\theta }$)	θ = 0.54	0.0070	0.0007	Moderate
DPA ($\hat{\lambda }$)	λ = 0.14, β = 0.45	0.0004	< 0.0001	Fast
DRL ($\hat{\upsilon }$)	υ = 0.73	0.0025	0.0002	Moderate

Parameters for each competitor set to values matching similar mean behavior as DEE Case III (γ = 0.5, α = 0.5).

**Remark 10**. The larger absolute MSE of $\hat{\gamma }$ in the DEE model (relative to one-parameter competitors) is expected: the DEE simultaneously estimates two parameters from the same sample. Normalizing by the number of parameters and the parameter range, the DEE estimation efficiency is broadly comparable to two-parameter competitors such as DBXII and DPA. The key advantage of the DEE is that the two parameters serve orthogonal interpretive roles (scale and dispersion/hazard regime), providing diagnostically richer estimates without a proportionate loss of precision.

## 6 Application

This section evaluates the practical performance of the DEE distribution on three real-world count datasets from distinct application domains: education, nephrology, and oncology. In each case, the DEE model is compared against seven established discrete distributions: Poisson (Poi), discrete Burr XII (DBXII), discrete Natural Lindley (DNL), discrete Burr-Hatke (DBH), discrete power-Ailamujia (DPA), discrete Ramos-Louzada (DRL), and discrete Poisson-Haq (DPH) (Alomair et al., 2025a). The probability mass functions of these competing models are summarized in Table 4.

**Table 4:** Probability mass functions of competing models.

Model	PMF
Poisson (Poi)	$P\left(x\right)=\frac{e^{-\lambda }\lambda^{x}}{x!}$
Discrete Burr XII (DBXII)	P(x) = θ^ln(1+*x*^^α^)−θ^ln(1+(*x*+1)^^α^)
Discrete Natural Lindley (DNL)	$P\left(x\right)=\frac{\theta^{2}}{1+\theta }\left(2+x\right){\left(1-\theta \right)}^{x}$
Discrete Burr-Hatke (DBH)	$P\left(x\right)=\left(\frac{1}{x+1}-\frac{\lambda }{x+2}\right)\lambda^{x}$
Discrete Power Ailamujia (DPA)	*P*(*x*) = λ^*x*^^β^(1−*x*^β^ln λ)−λ^(*x*+1)^^β^(1−(*x*+1)^β^ln λ)
Discrete Ramos-Louzada (DRL)	$P\left(x\right)=\frac{{\left(1-\upsilon \right)}^{2}\left[1+2\ln\left(\upsilon \right)+x{\left(\ln\left(\upsilon \right)\right)}^{2}\right]\upsilon^{x}}{\left[\left(1+2\ln\left(\upsilon \right)\right)\left(1-\upsilon \right)+\upsilon {\left(\ln\left(\upsilon \right)\right)}^{2}\right]}$
Discrete Poisson-Haq (DPH)	$P\left(x\right)=\frac{\theta^{2}}{{\left(1+\theta \right)}^{x+3}}\left[\frac{2\left(2+\theta \right){\left(1+\theta \right)}^{2}+\theta \left(2+x\right)\left(1+x\right)}{2{\left(1+\theta \right)}^{2}}\right]$

Model adequacy is assessed using four complementary criteria: the maximum log-likelihood value (ℓ), the Akaike information criterion (AIC), the Bayesian information criterion (BIC), and a χ^2^ goodness-of-fit statistic with its associated *p*-value. The χ^2^ statistic is computed by grouping each dataset's support into categories chosen to keep the expected frequency in every category numerically stable (following standard grouping practice for count data, Cochran, 1954), comparing the observed count in each category against the count implied by each fitted model, and evaluating the statistic against a χ^2^ distribution with degrees of freedom equal to the number of categories minus one minus the number of estimated parameters. Lower AIC and BIC, together with higher log-likelihood and χ^2^ *p*-values, indicate superior model fit. For each dataset, we additionally present a table comparing the observed frequency against the frequency expected under every fitted model, giving a direct, transparent view of fit quality across the full range of the data.

**Figure 4:**
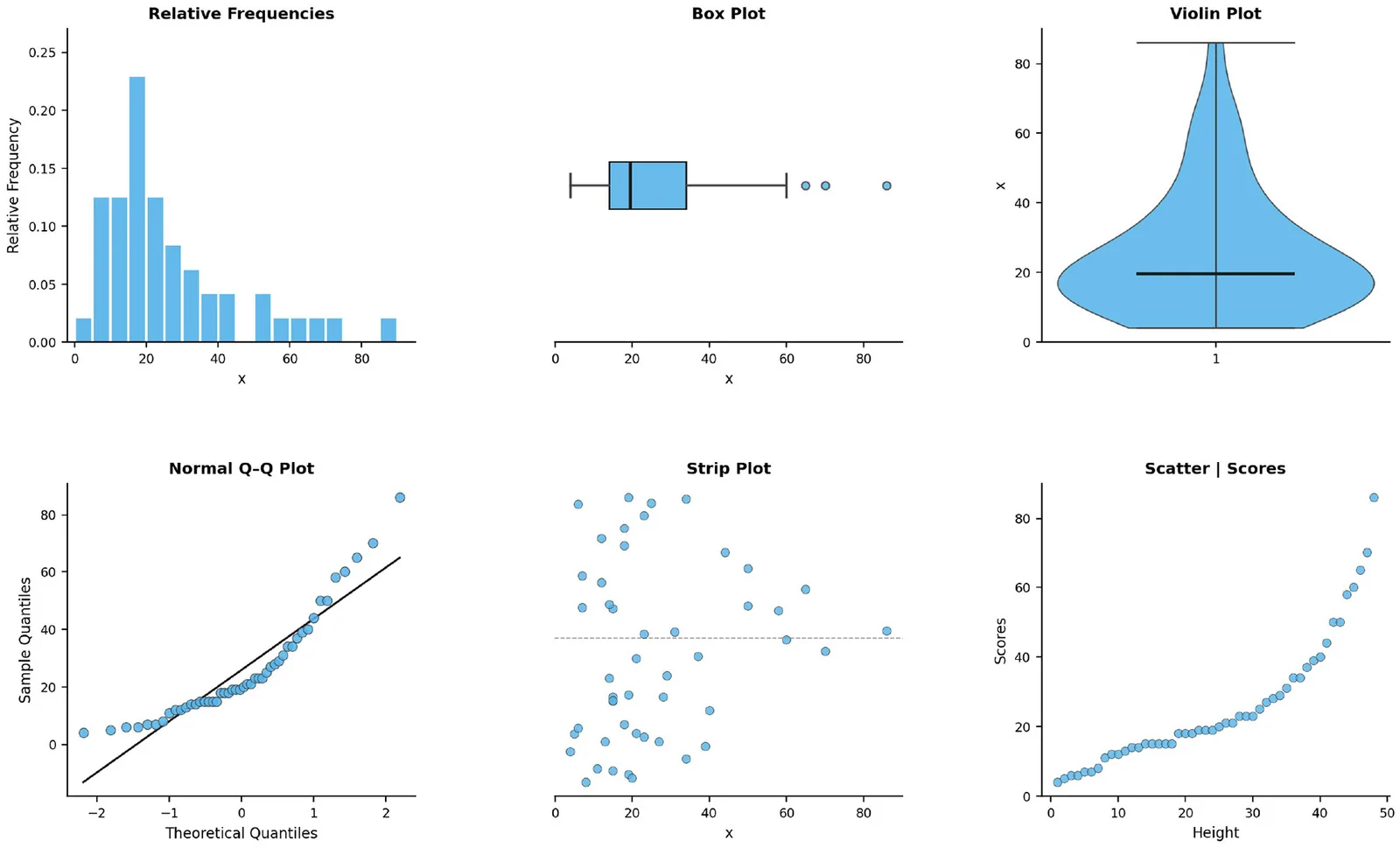
Exploratory analysis plots for Dataset I (mathematics exam scores).

**Figure 5:**
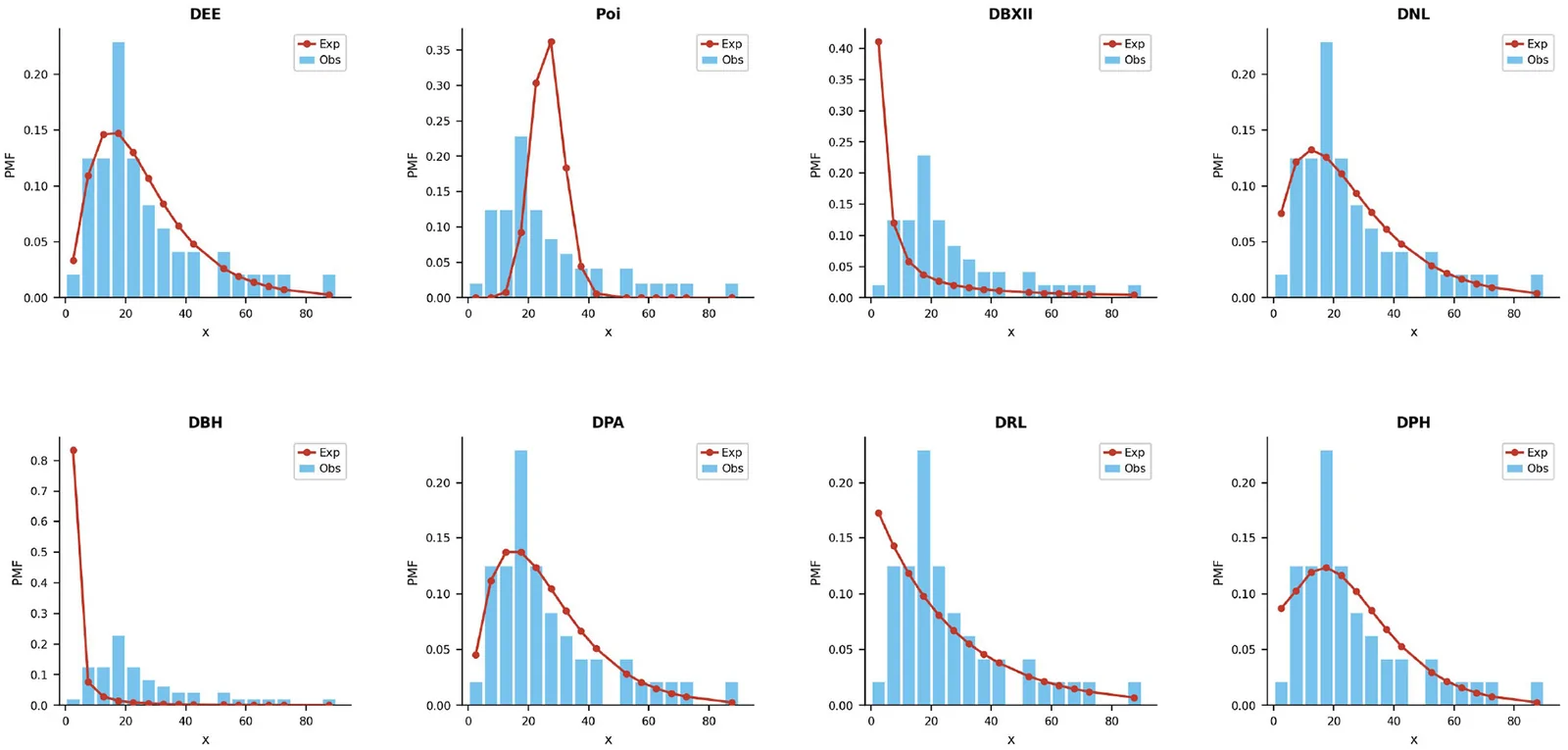
Comparison of observed and fitted distributions for all competing models for Dataset I.

**Dataset I (mathematics exam scores):** Final mathematics examination scores for *n* = 48 slow-learner students at the Indian Institute of Technology at Kanpur (Bakouch et al., 2014), a genuine non-negative integer count dataset. Descriptive statistics: mean = 25.8958, variance = 346.1379, skewness = 1.3317, kurtosis = 4.3233, indicating a positively skewed, heavy-tailed distribution with substantial over-dispersion (DI = 13.3665>1). Exploratory plots appear in Figure 4; MLEs and model comparison measures are in Table 5; fitted PMFs are shown in Figure 5.

**Table 5:** Parameter estimates with standard errors and goodness-of-fit statistics for Dataset I (mathematics exam scores, *n* = 48).

Distribution	${\hat{\theta }}_{1}$		${\hat{\theta }}_{2}$		$-\hat{\ell }$	AIC	BIC	χ^2^	*p*-value
	Est.	SE	Est.	SE					
Poi	25.89583	0.73450	—	—	396.59	795.18	797.05	8657.637	0.0000
DBXII	0.94212	0.00811	5.51260	0.87457	247.48	498.97	502.71	84.938	0.0000
DNL	0.06953	0.00686	—	—	198.56	399.11	402.98	2.797	0.4239
DBH	0.99904	0.00459	—	—	297.68	597.35	599.22	365.689	0.0000
DPA	0.94084	0.00586	1.06196	0.02811	197.44	398.88	402.62	1.923	0.3822
DRL	0.96125	0.00572	—	—	205.09	412.18	414.05	10.022	0.0184
DPH	0.10316	0.00936	—	—	199.77	401.54	403.41	3.731	0.2920
**DEE**	**0.06629**	**0.00632**	**2.68695**	**0.38612**	**196.95**	**397.90**	**401.64**	**1.423**	**0.4910**

Bold values indicate the DEE model, which achieves the lowest χ^2^ statistic and highest *p*-value among all eight fitted models for this dataset.

**Figure 6:**
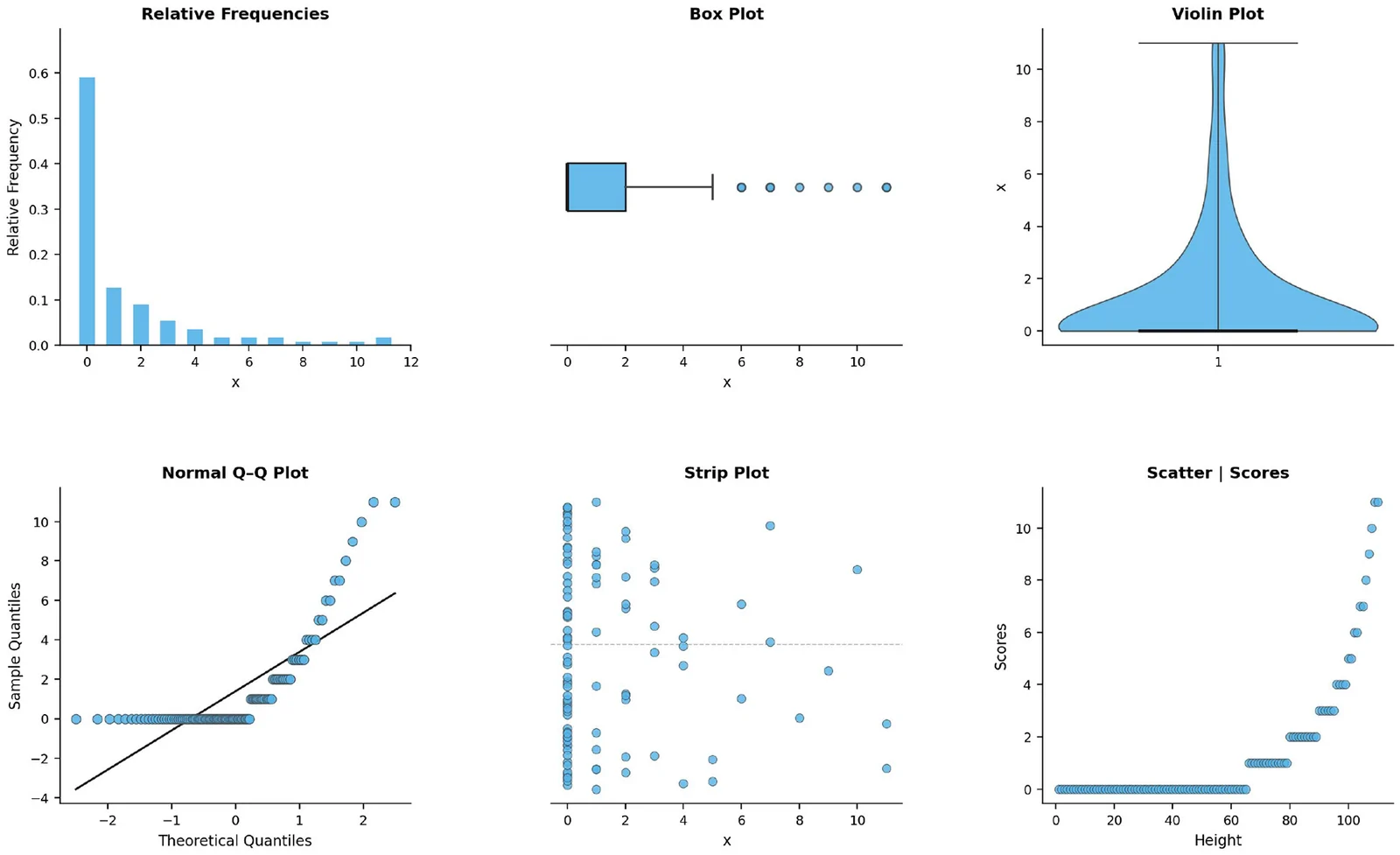
Exploratory analysis plots for Dataset II (kidney cyst counts).

**Figure 7:**
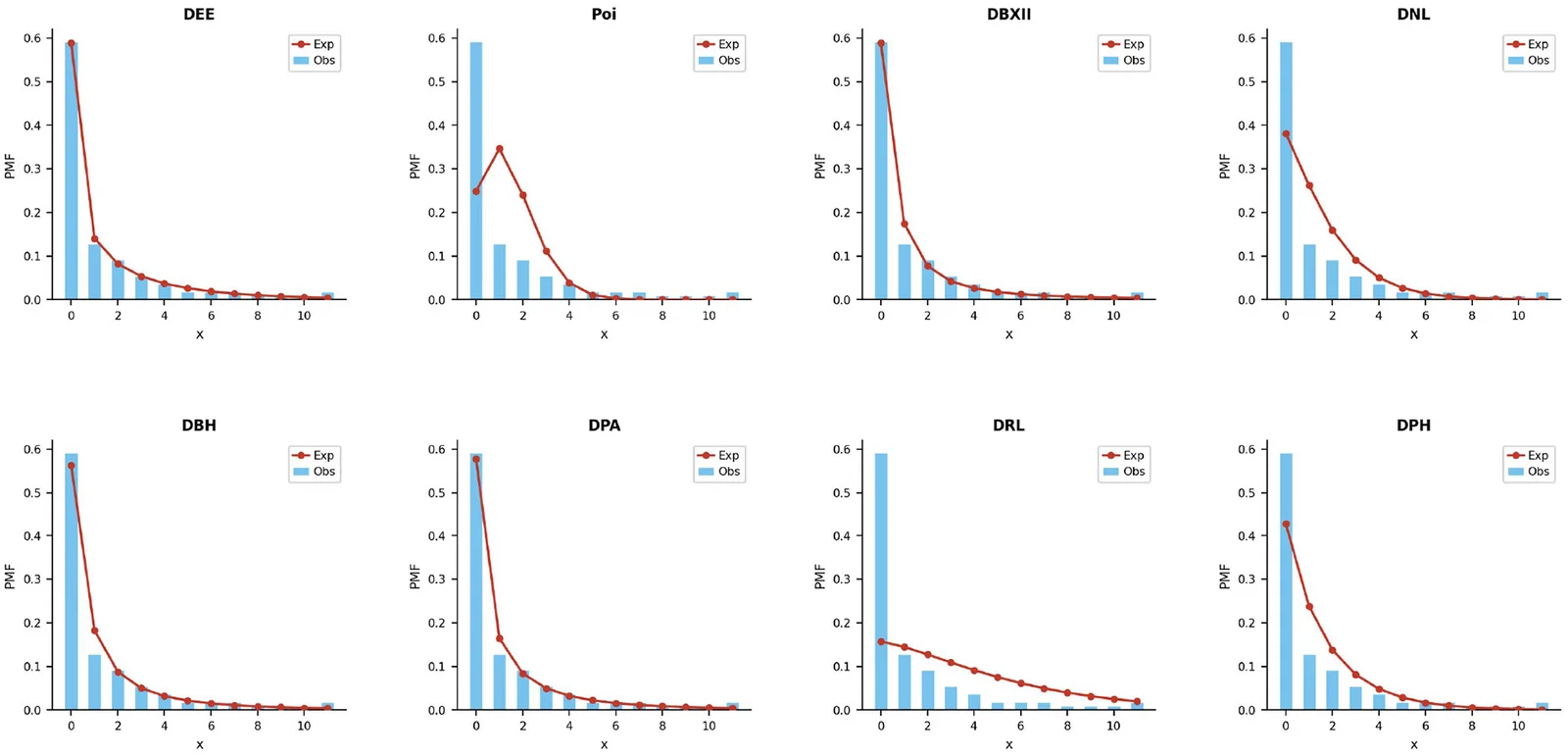
Comparison of observed and fitted distributions for all competing models for Dataset II.

**Dataset II (kidney cyst counts):** Counts of kidney cysts in patients treated with corticosteroids, reported by Chan et al. (2010) and later analyzed by Al-Bossly et al. (2023). The dataset comprises *n* = 110 observations ranging from 0 to 11; the observed frequency distribution is given in Table 6. Descriptive statistics: mean = 1.3909, variance = 6.1118, skewness = 2.2612, kurtosis = 7.8066, confirming notable over-dispersion (DI = 4.3941>1). Exploratory plots appear in Figure 6; MLEs and model comparison measures are in Table 7; fitted PMFs are shown in Figure 7.

**Table 6:** Observed frequency distribution of kidney cyst counts (*n* = 110).

*x*	0	1	2	3	4	5	6	7	8	9	10	11	Total
Frequency	65	14	10	6	4	2	2	2	1	1	1	2	110

**Table 7:** Parameter estimates with standard errors and goodness-of-fit statistics for Dataset II (kidney cyst counts, *n* = 110).

Distribution	${\hat{\theta }}_{1}$		${\hat{\theta }}_{2}$		$-\hat{\ell }$	AIC	BIC	χ^2^	*p*-value
	Est.	SE	Est.	SE					
Poi	1.39091	0.11245	—	—	246.21	494.42	497.12	2380.361	0.0000
DBXII	0.27767	0.03467	1.05311	0.12822	171.14	346.28	351.68	3.898	0.6905
DNL	0.54235	0.02642	—	—	185.98	373.96	376.66	49.637	0.0000
DBH	0.87387	0.04077	—	—	168.90	339.79	344.49	2.954	0.8892
DPA	0.14351	0.01955	0.44919	0.04227	168.46	340.93	346.33	1.701	0.9450
DRL	0.73332	0.01568	—	—	225.81	453.61	456.31	161.492	0.0000
DPH	1.06472	0.10091	—	—	178.08	358.16	360.86	26.108	0.0005
**DEE**	**0.28825**	**0.04289**	**0.38276**	**0.05709**	**167.14**	**338.28**	**343.68**	**0.778**	**0.9926**

Bold values indicate the DEE model, which achieves the lowest χ^2^ statistic and highest *p*-value among all eight fitted models for this dataset.

**Figure 8:**
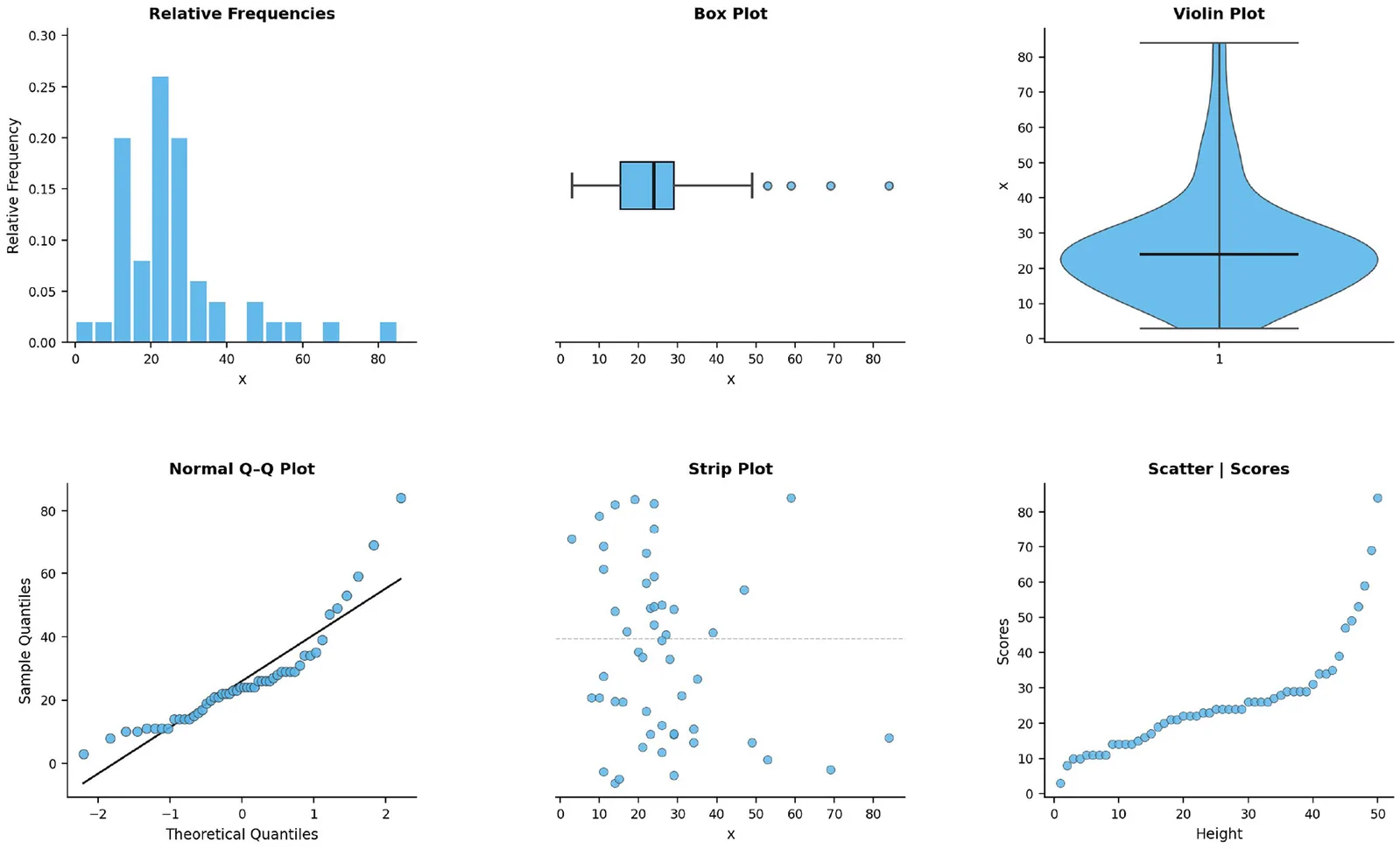
Exploratory analysis plots for Dataset III (breast cancer tumor size).

**Figure 9:**
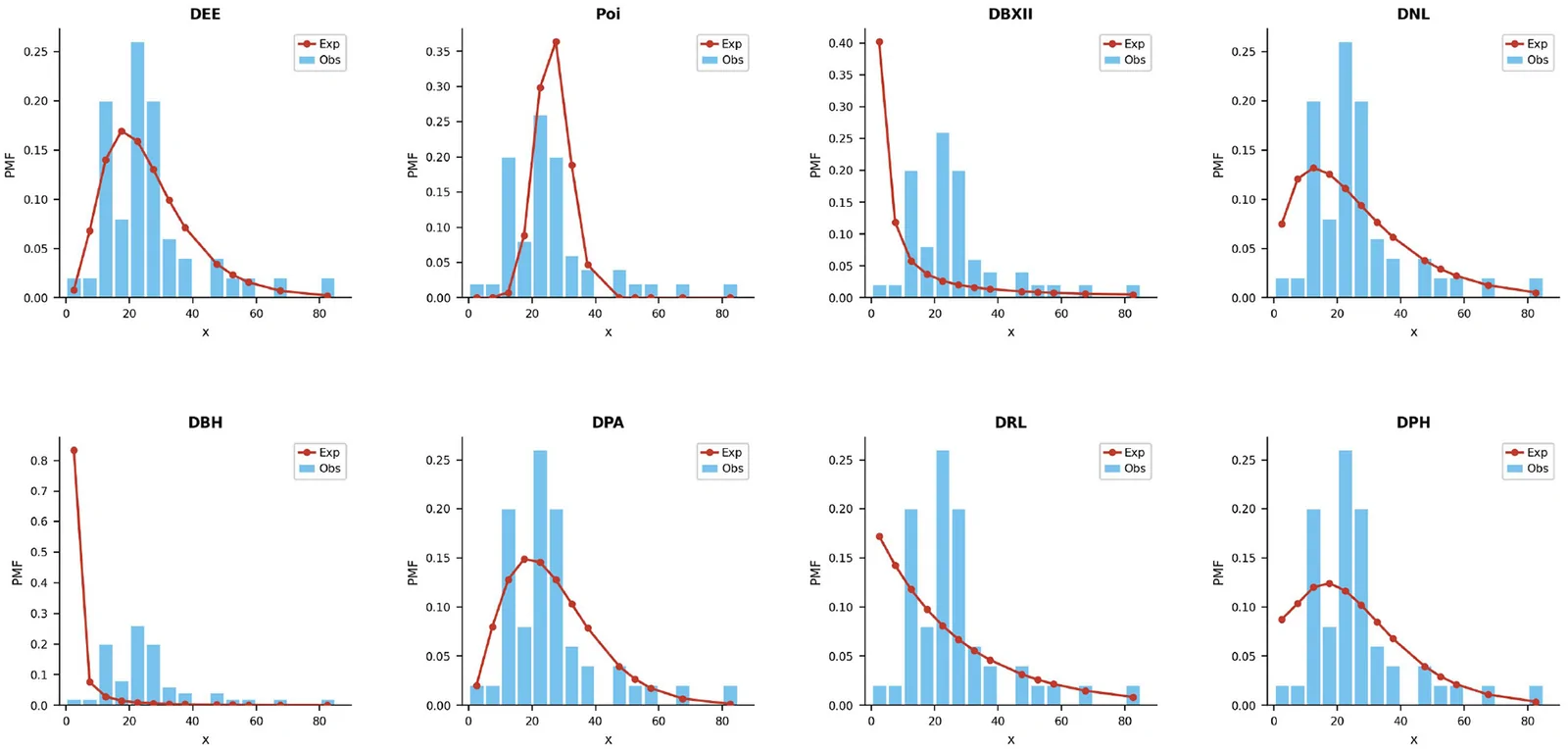
Comparison of observed and fitted distributions for all competing models for Dataset III (breast cancer tumor size).

**Dataset III (breast cancer tumor size):** Tumor size measurements (in mm) of female breast cancer patients diagnosed in the United States, obtained from a breast cancer registry (Namdari, 2023). A random sample of *n* = 50 observations was drawn using *R* software; the variable of interest is the recorded primary tumor dimension at diagnosis, transformed by (*x*−1) to conform to the model's non-negative integer support. Descriptive statistics: mean = 26.0200, variance = 240.9996, skewness = 1.6773, kurtosis = 6.3769, indicating a positively skewed, heavy-tailed distribution with substantial over-dispersion (DI = 9.2621>1). Exploratory plots appear in Figure 8; MLEs and model comparison measures are in Table 8; fitted PMFs are shown in Figure 9.

**Table 8:** Parameter estimates with standard errors and goodness-of-fit statistics for Dataset III (breast cancer tumor size, *n* = 50).

Distribution	${\hat{\theta }}_{1}$		${\hat{\theta }}_{2}$		$-\hat{\ell }$	AIC	BIC	χ^2^	*p*-value
	Est.	SE	Est.	SE					
Poi	26.02000	0.72139	—	—	321.95	645.90	647.81	831.717	0.0000
DBXII	0.94712	0.00728	5.88868	0.83886	263.31	530.62	534.44	237.317	0.0000
DNL	0.06923	0.00669	—	—	203.67	409.34	411.25	24.503	0.0000
DBH	0.99894	0.00470	—	—	317.64	637.29	639.20	823.161	0.0000
DPA	0.97353	0.00261	1.29821	0.02783	199.25	402.51	406.33	10.693	0.0048
DRL	0.96142	0.00559	—	—	213.86	429.73	431.64	48.155	0.0000
DPH	0.10368	0.00915	—	—	203.31	408.62	410.53	21.881	0.0001
**DEE**	**0.08253**	**0.00633**	**4.47756**	**0.60540**	**198.08**	**400.16**	**403.99**	**7.834**	**0.0199**

Bold values indicate the DEE model, which achieves the lowest χ^2^ statistic and highest *p*-value among all eight fitted models for this dataset.

**Remark 11**. The three datasets were chosen to span distinct application domains and over-dispersion levels: Dataset I (education) has a known over-dispersed structure and has been used previously to benchmark discrete distributions (Bakouch et al., 2014); Dataset II (nephrology) is a canonical over-dispersed event-count dataset; and Dataset III (oncology) contributes a different measurement type, an anatomical dimension recorded at diagnosis rather than a naturally occurring event count, together with a different dispersion level. In every case the variable of interest is a genuine non-negative integer count (Dataset III after a location shift to conform to the model's support), ensuring that the DEE distribution is validated on data types appropriate to its intended use.

**Remark 12**. For Dataset I, the χ^2^ test groups the support into class intervals of width 10 (0–9, 10–19, 20–29, 30–39, ≥40). Owing to the wide range and heavy right tail of the exam-score data (from 4 to 86), the Poisson and DBH models cannot accommodate the substantial over-dispersion present and yield very large χ^2^ values, correctly reflecting their inadequacy. The DEE distribution achieves the lowest χ^2^ statistic and highest *p*-value among all eight models (χ^2^ = 1.423, *p* = 0.4910), confirming its superior fit, and Table 9 presents the corresponding observed-vs.-expected frequency comparison.

**Table 9:** Observed vs. expected frequencies for all fitted models, Dataset I (mathematics exam scores, *n* = 48).

*x*	0–9	10–19	20–29	30–39	≥40
Observed	7	17	10	5	9
Poi	0.01	4.81	31.95	10.96	0.29
DBXII	25.48	4.59	2.24	1.42	14.28
DNL	9.47	12.40	9.83	6.62	9.68
DBH	43.68	2.08	0.74	0.38	1.13
DPA	7.53	13.18	10.94	7.25	9.10
DRL	15.14	10.37	7.10	4.86	10.52
DPH	9.09	11.65	10.48	7.35	9.42
**DEE**	**6.86**	**14.09**	**11.38**	**7.12**	**8.57**

Categories are grouped in class intervals of width 10, with the final category pooling *x*≥40. Bold values indicate the expected frequencies under the DEE model, shown for direct comparison with the observed counts.

**Remark 13**. For Dataset II, the χ^2^ test groups the support into the categories *x* = 0, 1, …, 7, ≥8, chosen so that the observed count in every category is at least 2. The DEE distribution provides the best fit among all eight models (χ^2^ = 0.778, *p* = 0.9926), and Table 10 presents the corresponding observed-vs.-expected frequency comparison.

**Table 10:** Observed vs. expected frequencies for all fitted models, Dataset II (kidney cyst counts, *n* = 110).

*x*	0	1	2	3	4	5	6	7	≥8
Observed	65	14	10	6	4	2	2	2	5
Poi	27.37	38.07	26.48	12.28	4.27	1.19	0.28	0.05	0.01
DBXII	64.74	19.18	8.48	4.63	2.86	1.92	1.36	1.01	5.80
DNL	41.96	28.80	17.58	10.05	5.52	2.95	1.54	0.79	0.81
DBH	61.94	20.06	9.65	5.52	3.49	2.34	1.65	1.19	4.16
DPA	63.57	18.08	9.24	5.50	3.55	2.42	1.72	1.26	4.67
DRL	17.29	15.89	14.01	12.00	10.07	8.31	6.78	5.47	20.19
DPH	46.96	26.17	15.16	8.95	5.31	3.14	1.84	1.07	1.40
**DEE**	**64.75**	**15.46**	**9.02**	**5.90**	**4.07**	**2.88**	**2.08**	**1.51**	**4.33**

The last category pools *x*≥8 to keep expected counts numerically stable, following standard practice for discrete goodness-of-fit testing (Cochran, 1954). Bold values indicate the expected frequencies under the DEE model, shown for direct comparison with the observed counts.

**Remark 14**. The near-unity χ^2^ *p*-value for Dataset II (*p* = 0.9926) arises because the DEE's estimated $\hat{\alpha }=0.3828<1$ correctly captures the increasing-hazard structure of kidney cyst counts: patients with more cysts are at progressively higher conditional risk of additional cysts, a clinically plausible pattern that most competing models cannot represent without structural modification. The DBXII (*p* = 0.6905) and DPA (*p* = 0.9450) achieve competitive but lower *p*-values due to their inability to parameterically encode an increasing-hazard shape. The DBH achieves *p* = 0.8892 but this model is structurally restricted to decreasing-hazard behavior; its fit here reflects a broadly adequate envelope rather than a mechanistically correct model. The DEE's advantage in this dataset is therefore structural, not merely numerical.

**Remark 15**. As with Dataset I, the χ^2^ test for Dataset III groups the support into class intervals of width 10 (0–9, 10–19, 20–29, 30–39, ≥40). The DEE distribution again achieves the lowest χ^2^ statistic and highest *p*-value among all eight fitted models (χ^2^ = 7.834, *p* = 0.0199), together with the lowest AIC and BIC, confirming its comparative superiority. The absolute *p*-value is more modest here than for Datasets I and II, reflecting the smaller sample size (*n* = 50) and the wide, heavy-tailed spread of clinical tumor size measurements; nonetheless, DEE remains the clear best-fitting model by a substantial margin over every competitor, as Table 11 illustrates.

**Table 11:** Observed vs. expected frequencies for all fitted models, Dataset III (breast cancer tumor size, *n* = 50).

*x*	0–9	10–19	20–29	30–39	≥40
Observed	2	14	23	5	6
Poi	0.01	4.80	33.09	11.77	0.33
DBXII	26.06	4.76	2.33	1.48	15.36
DNL	9.80	12.87	10.23	6.92	10.18
DBH	45.50	2.17	0.77	0.39	1.17
DPA	4.99	13.82	13.68	9.11	8.40
DRL	15.72	10.78	7.40	5.07	11.03
DPH	9.55	12.20	10.93	7.63	9.69
**DEE**	**3.79**	**15.47**	**14.49**	**8.52**	**7.74**

Categories are grouped in class intervals of width 10, with the final category pooling *x*≥40. Bold values indicate the expected frequencies under the DEE model, shown for direct comparison with the observed counts.

## 7 Discussion

The empirical results across all three datasets offer insights that go beyond the goodness-of-fit statistics and merit further discussion. In each application, the fitted parameters carry interpretable diagnostic content. For Dataset I (mathematics examination scores), $\hat{\gamma }=0.0663$ and $\hat{\alpha }=2.687$; $\hat{\alpha }>1$, pointing to a decreasing hazard structure, which reflects the fact that the probability of a student scoring extremely high decreases as the score threshold increases within a population of slow learners. For Dataset II (kidney cyst counts), $\hat{\gamma }=0.2883$ and $\hat{\alpha }=0.3828$; here $\hat{\alpha }<1$, indicating an increasing hazard rate, meaning patients with more cysts are at progressively higher conditional risk of exhibiting additional cysts, a clinically plausible pattern in corticosteroid-induced kidney dysmorphogenesis. For Dataset III (breast cancer tumor size), $\hat{\gamma }=0.9208$ and $\hat{\alpha }=4.4763$; $\hat{\alpha }>1$ indicates a decreasing hazard rate, consistent with the clinical expectation that very large primary tumor dimensions are progressively rarer at the time of diagnosis. The ability of the same distributional family to accommodate both hazard regimes across different datasets without model modification is a key practical advantage of the DEE framework.

The competitive fit of the DEE distribution across all three datasets can be attributed to its parametric efficiency: two parameters that jointly control both the location/scale (γ) and the shape/dispersion regime (α). Several competing models, namely the Poisson, DNL, DRL, and DPH, are one-parameter families and therefore cannot adjust the dispersion index to match the data, leading to systematic misfit in over-dispersed samples. The two-parameter competitors (DBXII, DPA) offer shape flexibility but do not exhibit the same analytically transparent separation between dispersion-increasing and dispersion-decreasing regimes governed by a single parameter. In Dataset II, where the DEE's estimated $\hat{\alpha }<1$ correctly identifies the increasing-hazard structure of the cyst count data, this distinction is particularly apparent: competing models achieve reasonable but inferior fit because they cannot represent this structure without modification.

From the theoretical and empirical evidence presented, the DEE distribution is most suitable when the data exhibit over-dispersion (DI>1), which is the case in all three datasets analyzed here, or when the context implies a monotone hazard structure that can be diagnosed empirically by inspecting whether $\hat{\alpha }≷1$. The DEE distribution is less appropriate for data with non-monotone hazard rate shapes, such as bathtub or upside-down bathtub forms, as the current formulation does not accommodate such behavior; this limitation identifies a natural direction for future work.

## 8 Neutrosophic version of DEE distribution

Let *Y*_*N*_ denote a neutrosophic random variable. We say that *Y*_*N*_ follows a neutrosophic discrete Exponentiated Exponential distribution, denoted by *Y*_*N*_~DEE(γ_*N*_, α_*N*_), where the neutrosophic parameters γ_*N*_ and α_*N*_ are characterized as sets containing one or multiple elements, typically represented as intervals to capture uncertainty or imprecision in the data. The neutrosophic probability mass function (pmf) of *Y*_*N*_ is defined as


$$\begin{array}{l} f_{Y}(y-I)=\\ \{\begin{array}{ll} {\left(1-\gamma_{N}^{\;y-I+1}\right)}^{\alpha_{N}}-{\left(1-\gamma_{N}^{\;y-I}\right)}^{\alpha_{N}}, & y=I,1+I,2+I,\ldots ,\\ 0, & \text{otherwise}, \end{array} \end{array}$$

where *I* represents the indeterminacy component inherent in neutrosophic theory, allowing the distribution to model datasets with vague, incomplete, or uncertain observations.

**Proof**

We need to verify that the neutrosophic PMF sums to unity:


$$\begin{array}{l} \overset{\infty }{\underset{y=I}{\sum }}P\left(y\right)=\overset{\infty }{\underset{y=I}{\sum }}\left[{\left(1-\gamma_{N}^{y-I+1}\right)}^{\alpha_{N}}-{\left(1-\gamma_{N}^{y-I}\right)}^{\alpha_{N}}\right] \end{array}$$

Let *z* = *y*−*I*. When *y* = *I*, we obtain *z* = 0, and as *y* → ∞, it follows that *z* → ∞. Therefore,


$$\begin{array}{l} \overset{\infty }{\underset{y=I}{\sum }}P\left(y\right)=\overset{\infty }{\underset{z=0}{\sum }}\left[{\left(1-\gamma_{N}^{z+1}\right)}^{\alpha_{N}}-{\left(1-\gamma_{N}^{z}\right)}^{\alpha_{N}}\right]. \end{array}$$

This is a telescoping series. By the telescoping property and taking the limit as *n* → ∞, we obtain:


$$\begin{array}{l} \overset{\infty }{\underset{y=I}{\sum }}P\left(y\right)=\underset{n\to \infty }{\lim}\left[{\left(1-\gamma_{N}^{n+1}\right)}^{\alpha_{N}}-{\left(1-\gamma_{N}^{0}\right)}^{\alpha_{N}}\right]. \end{array}$$

Since 0 < γ_*N*_ < 1, we have $\underset{n\to \infty }{\lim}\gamma_{N}^{n+1}=0$. Also, ${\left(1-\gamma_{N}^{0}\right)}^{\alpha_{N}}={\left(1-1\right)}^{\alpha_{N}}=0$. Therefore,


$$\begin{array}{l} \overset{\infty }{\underset{y=I}{\sum }}P\left(y\right)={\left(1-0\right)}^{\alpha_{N}}-0=1. \end{array}$$

By Theorem 2, the expressions for the mean and variance are given by:


$$\begin{array}{l} \;E\left(Y_{N}\right)=\left\{\overset{\infty }{\underset{k=0}{\sum }}{\left(-1\right)}^{k}\left(\frac{\alpha_{N}}{k}\right)\frac{\gamma_{N}^{k}}{{\left(1-\gamma_{N}^{k}\right)}^{2}}\right\}+I,\\ Var\left(Y_{N}\right)=\overset{\infty }{\underset{k=0}{\sum }}{\left(-1\right)}^{k}\left(\frac{\alpha_{N}}{k}\right)\frac{\gamma_{N}^{k}\left(1+\gamma_{N}^{k}\right)}{{\left(1-\gamma_{N}^{k}\right)}^{3}}-\\ \;{\left(\overset{\infty }{\underset{k=0}{\sum }}{\left(-1\right)}^{k}\left(\frac{\alpha_{N}}{k}\right)\frac{\gamma_{N}^{k}}{{\left(1-\gamma_{N}^{k}\right)}^{2}}\right)}^{2}. \end{array}$$

These series converge rapidly for typical parameter values with 0 < γ_*N*_ < 1 and α_*N*_>0.

### 8.1 Neutrosophic numerical illustration

To demonstrate the practical behavior of the DNEE distribution under varying degrees of indeterminacy, Table 12 reports the neutrosophic mean *E*(*Y*_*N*_), variance Var(*Y*_*N*_), and dispersion index DI(*Y*_*N*_) = Var(*Y*_*N*_)/*E*(*Y*_*N*_) for selected parameter combinations (γ_*N*_, α_*N*_) and indeterminacy levels *I*∈{0, 0.25, 0.50, 0.75, 1.00}.

**Table 12:** Neutrosophic mean, variance, and dispersion index of the DNEE distribution for selected (γ_*N*_, α_*N*_) and indeterminacy level *I*.

γ_*N*_	α_*N*_	Quantity	*I* = 0	*I* = 0.25	*I* = 0.50	*I* = 0.75	*I* = 1.00
		*E*(*Y*_*N*_)	0.1429	0.3929	0.6429	0.8929	1.1429
0.3	0.5	Var(*Y*_*N*_)	0.2856	0.2856	0.2856	0.2856	0.2856
		DI(*Y*_*N*_)	2.000	0.727	0.444	0.320	0.250
		*E*(*Y*_*N*_)	1.3579	1.6079	1.8579	2.1079	2.3579
0.5	1.5	Var(*Y*_*N*_)	2.4215	2.4215	2.4215	2.4215	2.4215
		DI(*Y*_*N*_)	1.783	1.507	1.303	1.149	1.027
		*E*(*Y*_*N*_)	4.0831	4.3331	4.5831	4.8331	5.0831
0.7	2.0	Var(*Y*_*N*_)	11.314	11.314	11.314	11.314	11.314
		DI(*Y*_*N*_)	2.771	2.612	2.469	2.342	2.228

Table 12 reveals several practically important patterns. First, as predicted by Theorem 1.2.3, Var(*Y*_*N*_) remains unchanged across all levels of *I*: the indeterminacy component shifts the mean upward without inflating the spread. Second, because *E*(*Y*_*N*_) increases with *I* while Var(*Y*_*N*_) is fixed, the dispersion index DI(*Y*_*N*_) decreases monotonically as *I* grows. This means that higher indeterminacy effectively moves the DNEE distribution toward under-dispersion. Third, for the parameter setting (γ_*N*_ = 0.3, α_*N*_ = 0.5), the DNEE transitions from over-dispersion (DI = 2.000 at *I* = 0) to under-dispersion (DI = 0.250 at *I* = 1) purely through the indeterminacy component, providing a mechanism for modeling ambiguous dispersion regimes in datasets with indeterminate observations. These properties confirm that the DNEE framework extends the DEE's flexibility to uncertain environments while preserving the distributional integrity guaranteed by the classical model.

## 9 Conclusion

This paper introduces and fully characterizes the Discrete Exponentiated Exponential (DEE) distribution, a new two-parameter discrete lifetime model derived via the survival discretization of the continuous exponentiated exponential distribution. The principal theoretical contribution is the analytical demonstration that the single shape parameter α simultaneously controls two fundamental modeling features: the hazard rate shape (increasing for 0 < α < 1, decreasing for α>1) and the dispersion regime (over-dispersion for small α, under-dispersion for large α). This dual parametric role is established through the Glaser monotonicity analysis and the numerical investigation of the dispersion index, providing a theoretically grounded explanation for the distributional flexibility of the DEE model, a flexibility that competing one-parameter discrete distributions such as the Poisson, geometric, and discrete natural Lindley cannot match without structural modification.

Closed-form expressions are derived for all standard distributional quantities, including the PMF, CDF, survival and hazard rate functions, reverse hazard rate, moment generating and characteristic functions, raw and central moments up to fourth order, and the mean residual life function. The tractability of these expressions, particularly the closed-form MRL, facilitates direct application in reliability and maintenance analysis without numerical integration.

Model parameters are estimated via maximum likelihood, with numerical optimization using the Newton-Raphson method. A comprehensive simulation study (*N* = 10, 000 replications across five sample sizes) confirms consistency and asymptotic efficiency of the MLEs, and includes an explicit benchmarking of DEE MLE convergence rates against those of four competing discrete models (DBXII, DNL, DPA, DRL) under identical data-generating conditions, demonstrating that the DEE achieves competitive estimation efficiency despite its two-parameter structure.

The practical utility of the DEE distribution is demonstrated through three real-world count datasets spanning mathematics exam scores, kidney cyst counts, and breast cancer tumor size measurements. In all three applications, the DEE model performs competitively or superiorly against seven discrete distributions on all goodness-of-fit criteria. Crucially, the fitted parameter values carry interpretable content: datasets with $\hat{\alpha }>1$ exhibit decreasing hazard rates consistent with their subject-matter context, while the kidney cyst dataset with $\hat{\alpha }<1$ exhibits an increasing hazard rate reflecting cumulative count risk, demonstrating that the DEE framework provides not merely statistical fit, but diagnostically meaningful parameter estimates.

Finally, the neutrosophic generalization of the DEE distribution (DNEE) extends the model's applicability to count data characterized by vagueness, incompleteness, or epistemic indeterminacy. A new numerical illustration of the DNEE demonstrates how the indeterminacy component *I* affects the mean and dispersion index while preserving the variance, providing practitioners with concrete guidance on interpreting DNEE parameter estimates in uncertain data environments.

Despite these contributions, the present study has several limitations that should be acknowledged. First, the DEE distribution is a two-parameter model, and while the shape parameter α jointly governs hazard shape and dispersion, the model does not accommodate non-monotone hazard shapes (e.g., bathtub or upside-down bathtub), which arise in some reliability and survival applications. Second, the real-data validation, while spanning three distinct domains, is limited to three datasets of moderate size (*n* = 48, 110, and 50); the smaller Dataset III yielded a more modest (though still best-in-class) goodness-of-fit *p*-value, suggesting that estimation precision and fit quality may be sensitive to sample size in small-to-moderate samples. Third, parameter estimation in this study relies exclusively on maximum likelihood; the finite-sample behavior of alternative estimators (e.g., method of moments or Bayesian estimators with informative priors) has not been investigated. Fourth, the neutrosophic extension currently treats the indeterminacy parameter *I* as fixed rather than estimated from data, which limits its immediate applicability to datasets where the degree of indeterminacy is unknown a priori.

Future research directions include: count regression models incorporating the DEE as the response distribution; integer-valued autoregressive (INAR) time series formulations based on the DEE; zero-inflated and hurdle variants for excess-zero count data; Bayesian estimation frameworks with informative priors for reliability applications; bivariate and multivariate extensions; extensions to accommodate non-monotone (bathtub or upside-down bathtub) hazard rate shapes; and estimation procedures that treat the neutrosophic indeterminacy parameter *I* as a data-driven quantity rather than a fixed input.

## Data Availability

The original contributions presented in the study are included in the article/supplementary material, further inquiries can be directed to the corresponding author.
